# Respiratory complex I is essential to induce a Warburg profile in mitochondria-defective tumor cells

**DOI:** 10.1186/2049-3002-1-11

**Published:** 2013-03-18

**Authors:** Claudia Calabrese, Luisa Iommarini, Ivana Kurelac, Maria Antonietta Calvaruso, Mariantonietta Capristo, Pier-Luigi Lollini, Patrizia Nanni, Christian Bergamini, Giordano Nicoletti, Carla De Giovanni, Anna Ghelli, Valentina Giorgio, Mariano Francesco Caratozzolo, Flaviana Marzano, Caterina Manzari, Christine M Betts, Valerio Carelli, Claudio Ceccarelli, Marcella Attimonelli, Giovanni Romeo, Romana Fato, Michela Rugolo, Apollonia Tullo, Giuseppe Gasparre, Anna Maria Porcelli

**Affiliations:** 1 Dip. Scienze Mediche e Chirurgiche (DIMEC), U.O. Genetica Medica, Pol. Universitario S. Orsola-Malpighi, Università di Bologna, via Massarenti 9, Bologna, 40138, Italy; 2 Dip. Farmacia e Biotecnologie (FABIT), Università di Bologna, via Belmeloro 6, Bologna, 40126, Italy; 3 Dip. Medicina Specialistica, Diagnostica e Sperimentale, Università di Bologna, Viale Filopanti 22, Bologna, 40126 and via Massarenti 9, 40138, Italy; 4 Laboratorio di Oncologia Sperimentale, Istituti Ortopedici Rizzoli, Bologna, 40136, Italy; 5 Dip. Scienze Biomediche, Università di Padova, Padova, Italy; 6 Istituto di Tecnologie Biomediche - ITB, Consiglio Nazionale delle Ricerche (CNR), Bari, Italy; 7 Dip. Bioscienze, Biotecnologie e Scienze Farmacologiche, Università di Bari, via E.Orabona 4, Bari, 70126, Italy; 8 IRCCS Istituto delle Scienze Neurologiche, Bologna, 40100, Italy; 9 Dip. Sc. Radiologiche ed Istopatologiche, Pol. Universitario S.Orsola-Malpighi, Università di Bologna, via Massarenti 9, Bologna, 40138, Italy; 10 Centro Interdipartimentale di Ricerca Industriale Scienze della Vita e Tecnologie per la Salute, Università di Bologna, Bologna, 40100, Italy

**Keywords:** Complex I, mtDNA mutation, HIF-1α, Mitochondria, Cancer, Warburg effect, Allotopic expression

## Abstract

**Background:**

Aerobic glycolysis, namely the Warburg effect, is the main hallmark of cancer cells. Mitochondrial respiratory dysfunction has been proposed to be one of the major causes for such glycolytic shift. This hypothesis has been revisited as tumors appear to undergo waves of gene regulation during progression, some of which rely on functional mitochondria. In this framework, the role of mitochondrial complex I is still debated, in particular with respect to the effect of mitochondrial DNA mutations in cancer metabolism. The aim of this work is to provide the proof of concept that functional complex I is necessary to sustain tumor progression.

**Methods:**

Complex I-null osteosarcoma cells were complemented with allotopically expressed complex I subunit 1 (MT-ND1). Complex I re-assembly and function recovery, also in terms of NADH consumption, were assessed. Clones were tested for their ability to grow in soft agar and to generate tumor masses in nude mice. Hypoxia levels were evaluated via pimonidazole staining and hypoxia-inducible factor-1α (HIF-1α) immunoblotting and histochemical staining. 454-pyrosequencing was implemented to obtain global transcriptomic profiling of allotopic and non-allotopic xenografts.

**Results:**

Complementation of a truncative mutation in the gene encoding *MT-ND1,* showed that a functional enzyme was required to perform the glycolytic shift during the hypoxia response and to induce a Warburg profile *in vitro* and *in vivo*, fostering cancer progression. Such trigger was mediated by HIF-1α, whose stabilization was regulated after recovery of the balance between α-ketoglutarate and succinate due to a recuperation of NADH consumption that followed complex I rescue.

**Conclusion:**

Respiratory complex I is essential for the induction of Warburg effect and adaptation to hypoxia of cancer cells, allowing them to sustain tumor growth. Differently from other mitochondrial tumor suppressor genes, therefore, a complex I severe mutation such as the one here reported may confer anti-tumorigenic properties, highlighting the prognostic values of such genetic markers in cancer.

## Background

Aerobic glycolysis is one of the main hallmarks of cancer cells. The seminal observation by Otto Warburg at the beginning of last century [1,2] has fostered compelling efforts in elucidating the profound metabolic changes that transformed cells undergo from initial tumor to the spread of metastases, which to date remain largely obscure. It is still unclear why highly proliferative and invasive cancer cells settle for an energetically less efficient aerobic glycolysis, rather than oxidative phosphorylation (OXPHOS), which generates far more ATP from a single glucose molecule. The shift toward glycolysis has been ascribed to four potential causes, namely (i) a mitochondrial dysfunction, which forces cells to rely on glycolysis, (ii) an upregulation of glycolytic genes driven by the activation of oncogenes, (iii) the triggering of a strong hypoxic response that turns down oxygen-dependent respiration and (iv) the redirection of glucose catabolism towards macromolecular biosynthesis [3,4].

The classical Warburg hypothesis has recently been revisited, as tumors with active mitochondrial metabolism have been identified, indicating that aerobic glycolysis does not necessarily involve a decrease in mitochondrial function [5]. It is likely that glycolytic and oxidative metabolisms interchange in a wave pattern during the proliferation and transformation of cancer cells, constantly subjected to selective pressures in terms of nutrients and oxygen availability, determined by the ever-changing tumor microenvironment [6]. In this context, mitochondria might represent the main hub for the wave-like regulation of cancer metabolism [7]. In fact, they host a number of essential metabolic processes, such as the oxygen-dependent ATP production, redox regulation and biosynthetic reactions [8]. Moreover, mitochondria are involved in the regulation of hypoxic adaptation [9], a process that must be triggered in any solid neoplasia to overcome the initial steps of tumorigenesis and is controlled by hypoxia-inducible factor 1α (HIF-1α) [10].

During O_2_ deprivation, HIF-1α stabilization and activation is needed for cells to respond to hypoxia, upregulating the expression of pivotal glycolytic genes [11]. Therefore, in this condition, HIF-1α increases glycolysis and suppresses the tricarboxylic acids (TCA) cycle and, ultimately, mitochondrial respiration [12,13]. HIF-1α stabilization is tightly controlled by prolyl-hydroxylases (PHDs), enzymes whose HIF-1α-degrading activity is allosterically regulated by α-ketoglutarate (α-KG) and succinate (SA), two key metabolites of the TCA cycle, which feeds reducing equivalents to the respiratory chain for ATP production. In this frame, we have recently shown that a dysfunction in complex I (CI), caused by the severe m.3571insC/*MT-ND1* mitochondrial DNA (mtDNA) mutation, strongly contributes to HIF-1α destabilization and lack of hypoxia adaptation, which eventually leads to an antitumorigenic effect [14,15]. Complex I is the largest and least understood component of the respiratory chain, which catalyzes the transfer of electrons from NADH to flavin mononucleotide and then to ubiquinone [16]. The holoenzyme function is necessary for ATP production and for the maintenance of cellular redox state, such as NAD^+^/NADH ratio and reactive oxygen species (ROS) levels [17]. Complex I dysfunction, specifically that induced by the occurrence of mutations in respiratory genes encoded within the multicopy mtDNA, has generally been described as pro-tumorigenic [18,19]. An open debate scans the evidence that mtDNA mutations, according to their overall effect on the respiratory chain and, indirectly, on other oxidative metabolism pathways, may not behave univocally as pro-tumorigenic and pro-metastatic events [15,20]. Mutations in CI genes encoded within the mtDNA, in fact, have been shown both to foster and inhibit the replication of tumors in which they occur, a two-sided effect that depends on the percentage of mutant mtDNA copies (heteroplasmy) and on the capacity of the mutation to disassemble CI [21]. Therefore, it is likely that CI may no longer be looked upon as an enzyme that merely contributes to mitochondrial function. Nevertheless, although its role in determining the fate of cancer cells upon deregulation of their metabolic switch during tumor progression is considered increasingly crucial, it remains ambiguous. Furthermore, the molecular mechanisms through which CI may regulate metabolic adaptation in tumorigenesis still remain practically uninvestigated.

In the attempt to assign a role to CI within the Warburg effect, we have demonstrated that this mitochondrial enzyme is required in order to perform the metabolic switch towards glycolysis during the hypoxia response, a pivotal goal in the metabolic reprogramming of cancer cells.

## Methods

### Cell lines

In this study, 143B osteosarcoma-derived cybrids harboring the homoplasmic m.3571insC mutation in *MT-ND1*[HGCN:7455], their counterpart OS-93^ND1^, allotopically complemented with wild-type *MT-ND1* and their wild-type mtDNA counterpart (CC) were used. Mock cells transfected with empty vector are indicated as OS-93. Cybrids were obtained and cultured as previously described [15].

### Nucleic acid extraction

DNA from snap-frozen xenografts and cultured cells (7 × 10^5^) was extracted with GenElute™ Mammalian Genomic DNA Miniprep Kit (Sigma-Aldrich, Milan, Italy). RNeasy Plus Mini kit (Qiagen, Milan, Italy) was used to extract RNA from snap-frozen xenografts. Cell lines RNA was obtained using TRIzol reagent (Invitrogen, Milan, Italy) and following manufacturer’s instructions.

### *MT-ND1* allotopic construct

Wild-type *MT-ND1* was cloned from cDNA of TPC1 cells derived from a papillary thyroid carcinoma [22] and inserted into a p3XFLAG-CMV™-14 expression vector (Sigma-Aldrich, Milan, Italy). The *MT-ND1* sequence was identical to the mtDNA revised Cambridge Reference Sequence (rCRS) [GenBank:NC_012920.1]. FLAG epitope was excluded from the transgene frame in order not to affect protein folding. *COX10* [GenBank:U09466] 3^′^- and 5^′^-UTR were cloned according to Bonnet *et al*. [23]. The 5^′^-UTR from nuclear-encoded mitochondrial protein COX10 was cloned upstream of *nND1* for mRNA targeting to the mitochondria outer membrane, along with mitochondrial targeting sequence (N-terminal MTS). The 3^′^-UTR from *COX10* was inserted downstream of *nND1* in order to ensure mRNA stability. Site-directed *in vitro* mutagenesis was performed with the QuikChange Lightning Multi Site-Directed Mutagenesis Kit (Strategene, Agilent Technologies, Santa Clara, CA, USA) according to the manufacturer’s instructions. Oligonucleotides designed for this purpose are available on request.

### Cell transfection, selection and assessment of allotopic MT-ND1 expression

Cells were transfected with p3XFLAG-CMV™-14 empty vector and with the nND1 allotopic construct by using Lipofectamine 2000 transfection reagent (Invitrogen, Milan, Italy) following the manufacturer’s protocol. Stable clones were obtained by selection with 400 μg/mL G418 (Invitrogen, Milan, Italy) and the antibiotic resistant clones were double-selected by growing them in DMEM without glucose supplemented with 5 mM galactose, 5 mM Na-pyruvate and 10% FBS, in order to eliminate false positive clones. The expression of allotopic ND1 was assessed by quantitative real-time PCR (qRT-PCR). Total RNA was extracted from OS-93 and OS-93^ND1^ clones and 1 μg was used for retrotranscription with Transcriptor First Strand cDNA Synthesis Kit (Roche Diagnostics, Monza, Italy), by using random hexameric primers. Primer and TaqMan® probes sequences were designed using Primer3 software [24] and the presence of 3′ intra-/inter-primer similarity was ruled out using IDT OligoAnalyzer tool [25]. Sequences are available upon request. Allotopic nND1 PCR product spanned the region between the *COX10* MTS and 5^′^-*nND1*, in order to exclude any endogenous transcript, and the normalization was performed on pCMV levels, which is present in plasmid DNA but not translated, in order to exclude plasmid DNA contamination. Human ACTB gene[GenBank:M28424] was used as reference gene. The PCR reaction was performed with LightCycler® 480 Probes Master and run in LightCycler®480 Real-Time PCR System (Roche Diagnostics, Monza, Italy), using the following conditions: 95°C, 5 minutes; 45 cycles of 95°C, 15 sec, and 60°C, 45 sec. Absolute quantification was performed using a standard curve prepared by serial dilutions of purified p3XFLAG-CMV™-14 containing the allotopic construct.

### mtDNA sequencing and low heteroplasmy detection

Whole mtDNA resequencing was performed as previously described [26] in order to verify that xenografts had not accumulated mutations apart from the m.3571insC. Mutant load of m.3571insC was determined using fluorescent PCR (F-PCR) and denaturing high performance liquid chromatography (DHPLC) according to previously optimized protocols for mutations in difficult sequence contexts such as homopolymers [27]. Each analysis was performed in triplicate.

### SDS-PAGE

Mitochondrial enriched fraction was obtained by the subcellular fractionation in the presence of digitonine (50 μg/mL) [28]. Total lysates were prepared from 30 mg of xenografts as previously described [15]. Mitochondrial proteins (40 μg) or total xenograft and cell lysates (80 μg) were separated by 10% SDS-PAGE and transferred onto nitrocellulose membrane as previously reported [15].

### Western blot

Nitrocellulose membranes were incubated with antibodies against voltage-dependent anion channel (VDAC) (1:1000, BioVision, Mountain View, CA, USA), ND1 (1:1000, a gift from A. Lombes, Unite de Recherche INSERM 153, Hospital de la Salpetriere, Paris, France), HIF-1α (1:1000, Bethyl Laboratories, Montgomery, TX, USA), LDHA (1:1000, Sigma-Aldrich, Milan, Italy). Secondary antibodies used were peroxidase-conjugated anti-mouse or anti-rabbit (1:2000, Jackson ImmunoResearch, Suffolk, England). Chemiluminescence signals were obtained with Kodak molecular imaging apparatus (Kodak, Rochester, NY, USA). Coomassie staining was used as the loading control.

### Complex I In-Gel-Activity (CI-IGA) assay

Mitochondrial enriched fractions (for sample preparation see section SDS-PAGE) were solubilized with n-dodecyl-maltoside (2.5 g/g protein) as previously reported [29]. Proteins (100 μg) were separated by 4 to 13% Blue Native gradient gel (BN-PAGE) and CI-IGA was detected as previously described [30].

### NAD^+^/NADH ratio determination

Aliquotes of 1.5 × 10^6^ cells were washed and resuspended in 1 mL of ice-cold PBS and extracted for NADH and NAD^+^ determination. For NADH analysis, cell suspension was treated with 0.5 M potassium hydroxide containing 50% (v/v) ethanol and 35% (w/v) cesium chloride, immediately cooled on ice, centrifuged at 4°C to remove insoluble material and the supernatant (100 μL) was injected on C18 column. For NAD^+^ determination, cell suspension was treated with 1 M perchloric acid, immediately cooled on ice, and centrifuged at 4°C to remove insoluble material. Perchloric acid was neutralized with potassium hydroxide and centrifuged immediately before injection. The supernatant was injected (100 μL) on C18 column. The pyridine nucleotides were extracted and detected as described [31] on a Kinetex reversed phase C18 column (250 × 4.6 mm, 5 μm; Phenomenex, Torrance, CA, USA), with a two-pump Waters 510 system equipped with a variable volume injector*.* Absorbance at 260 nm for NAD^+^ and at 340 nm for NADH was monitored by a photodiode array detector (Waters 996). NADH and NAD^+^ peaks were identified by comparison of their retention times with those of standards and confirmed by co-elution with standards. The quantification was obtained from peak area measurement compared to standard curves.

### ATP synthesis measurement

The rate of mitochondrial ATP synthesis driven by CI and CII was performed in aliquots of digitonin-permeabilized cells and normalized on citrate synthase (CS) activity as previously described [32]. Briefly, aliquots (0.1 to 0.2 mg protein) were incubated with 5 mM malate plus 5 mM pyruvate (CI substrates) or with 10 mM succinate (CII substrate) plus 2 μg/mL rotenone. The reaction was started by addition of 0.2 mM ADP in the presence of luciferine/luciferase, and chemiluminescence was evaluated as a function of time with a luminometer. After addition of 10 μM oligomycin, the chemiluminescence signal was calibrated with an internal ATP standard.

### Oxygen consumption rate (OCR)

OCR in adherent cells was measured with an XF24 Extracellular Flux Analyzer (Seahorse Bioscience, Billerica MA, USA). Cells were seeded in XF24 cell culture microplates at 3 × 10^4^ cells/well in 200 μL of DMEM containing 4.5 g/L glucose and incubated at 37°C in 5% CO_2_ for 24 h. Assays were performed as previously reported [32]. Data are expressed as pmoles of O_2_ per minute per 3 × 10^4^ cells.

### Mitochondrial membrane potential determination

Cells (3 × 10^5^) were seeded onto 24 mm-diameter round glass coverslips and grown for 2 days. Mitochondrial membrane potential (ΔΨ_m_) was measured by the accumulation of tetramethylrhodamine methyl esther (TMRM) as previously reported [30]. Data were acquired and analyzed using MetaFluor software (Universal Imaging Corp., Downington, PA, USA). Clusters of several mitochondria were identified as regions of interest, and fields not containing cells were taken as background. Sequential digital images and fluorescence intensity were acquired every minute. Fluorescence values were obtained by subtracting background values from those of corresponding mitochondrial areas of interest, for each time point, and expressed as percentage of T0 (100%).

### Soft agar

Anchorage-independent cell growth was determined in 0.33% agarose with a 0.5% agarose underlayer. Cell suspensions (2 × 10^4^ cells) were plated in semisolid medium, in absence or presence of 1 μM dimethyloxallylglycine (DMOG). Colonies were counted after 7 days at a magnification of 10× with an inverted microscope (Nikon Diaphot, Nikon Instruments, Florence, Italy). Plate pictures and magnifications were obtained with a Kodak molecular imaging apparatus (Kodak, Rochester, NY, USA). Effects of DMOG on HIF-1α stabilization were validated by western blotting (Additional file 1: Figure S1). The index of colony forming ability was calculated as the ratio between untreated OS-93^ND1^ and OS-93 cells and then used for normalization of DMOG-treated cells. The *t*-test was used for statistical comparison.

### *In vivo* tumor growth analysis

Cells (3 × 10^6^) were suspended in 0.2 mL sterile PBS and injected subcutaneously in 4 to 7 week-old athymic Crl:CD-1-*Foxn1*^*nu/nu*^ mice (referred to as nude mice, purchased from Charles River, Lecco, Italy). Experiments were authorized by the institutional review board of the University of Bologna and performed according to Italian and European guidelines. Individually tagged virgin female mice (10 per experimental group) were used. Tumor growth was assessed with a caliper; volume was calculated as:

$$\Pi {\left(\surd \left(a*b\right)\right)}^{3}/6$$

where a = maximal tumor diameter, and b = tumor diameter perpendicular to a.

### Electron microscopy

Xenograft biopsies were immediately collected and processed as previously described [15]. Samples were observed with a JEM-1011 Transmission Electron Microscope (Jeol Ltd, Milan, Italy).

### cDNA library preparation and mRNA sequencing

Ultradeep pyrosequencing was performed using 454 GS FLX Titanium platform (Roche Diagnostics, Monza, Italy). RNA quality was assessed by a 2100 Bioanalyzer (Agilent Technologies, Santa Clara, CA, USA). Total RNA from each sample (5 to 8 μg) was used for poly(A) mRNA selection, using Oligotex mRNA kit (Qiagen, Milan, Italy). The poly(A)-enriched RNA samples were reverse-transcribed into cDNA using random-sequence primers. cDNA libraries preparation and subsequent pyrosequencing (applying 200-nucleotide cycles) were carried out according to the manufacturer’s instructions.

### RNA-Seq data analysis

RNA-seq reads obtained (Additional file 2: Table S1) were tested for sequence quality by FastQC [33] (Additional file 3: Figure S2) and those ≥100 bp were mapped onto the hg18/NCBI36-annotated human genome, using the Next Generation Sequencing. TRanscriptome profile Explorer (NGS-Trex) platform [34]. Multiple mappings were allowed to avoid too large a cutoff derived from paralogous genes (Additional file 4; Methods). Genes were considered expressed if at least one read was mapped. Raw digital expression read count per gene (considering all the mapped mRNA isoforms together) was used for the differential expression analysis with the edgeR [35] package of Bioconductor [36] (Additional file 4; Methods). Only genes with at least one read in all the four samples studied were considered in the statistical analysis using Fisher’s exact test. A *P*-value ≤0.05 was used as the threshold to consider a gene as differentially expressed (DE). The Bioconductor goseq [37] package was used to associate all DE genes to Gene Ontology (GO) categories and Kyoto Encyclopedia of Genes and Genomes (KEGG) pathways (Additional file 4; Methods), while the GeneMania server [38] was used to carry out gene network analyses by searching among several interaction databases, related bibliographic references and for GO categories enrichment within only overexpressed genes found in each group. The HIF-1α pathway reconstruction was manually curated [39-44] from interaction databases (BioGRID, HPRD, Pathway Commons, GEO dataset), with GeneMania and from NCBI Interaction and GeneRIF sections of the *HIF-1A* gene entry [45]. Representation of upregulated and downregulated genes in the heatmaps was prepared using the limma package of R [46], applying a hierarchical clustering onto log_2_ normalized digital gene expression values (Additional file 4; Methods). Validation of RNA-Seq data was performed with qRT-PCR as indicated in Additional file 4; Methods.

### Immunohistochemical analysis

Immunohistochemical (IHC) analysis with antibodies against NDUFB8 subunit of CI (Invitrogen, Milan, Italy) and HIF-1α (Upstate Biotech, Billerica, MA, USA) was performed as previously reported [47]. The semi-quantitative analysis of the stained sections was done by light-microscopy at 100× magnification. The evaluation of cytoplasmic NDUFB8 immunostaining was performed according to the percentage of positive cells (range 0 to 4) and to the staining intensity (range 0 to 3) using a modified immunoreactivity score (IRS) [48,49]. The final staining evaluation for each sample was obtained by combining the two values (range 0 to 12). The population of cells with a positive HIF-1α nuclear immunostaining was quantified using a computerized, morphometric, interactive, digital image analysis as previously described [50]. The labeling index was expressed as the percentage of the labeled nuclear area over the total nuclear area for tumor cells in the section.

### Pimonidazole staining

Animals were injected intraperitoneally with 60 mg/kg pimonidazole (Hypoxyprobe-1 Plus Kit, HPI, Burlington, MA, USA) 3 h prior to sacrifice. Xenografts were treated and fluorescence visualized as previously reported [15].

### α-KG and SA measurements

Measurements of metabolites α-KG and SA were carried out in *ex vivo* cell lines derived from OST-93 and OST-93^ND1^ essentially as previously described [15]. The *t*-test was used for statistical comparison.

### Statistical analyses

Analysis of variance (ANOVA) was used for all statistical analyses unless otherwise indicated.

## Results

### Allotopic *MT-ND1* expression corrects CI-dependent mitochondrial energetic dysfunction

**Figure 1:**
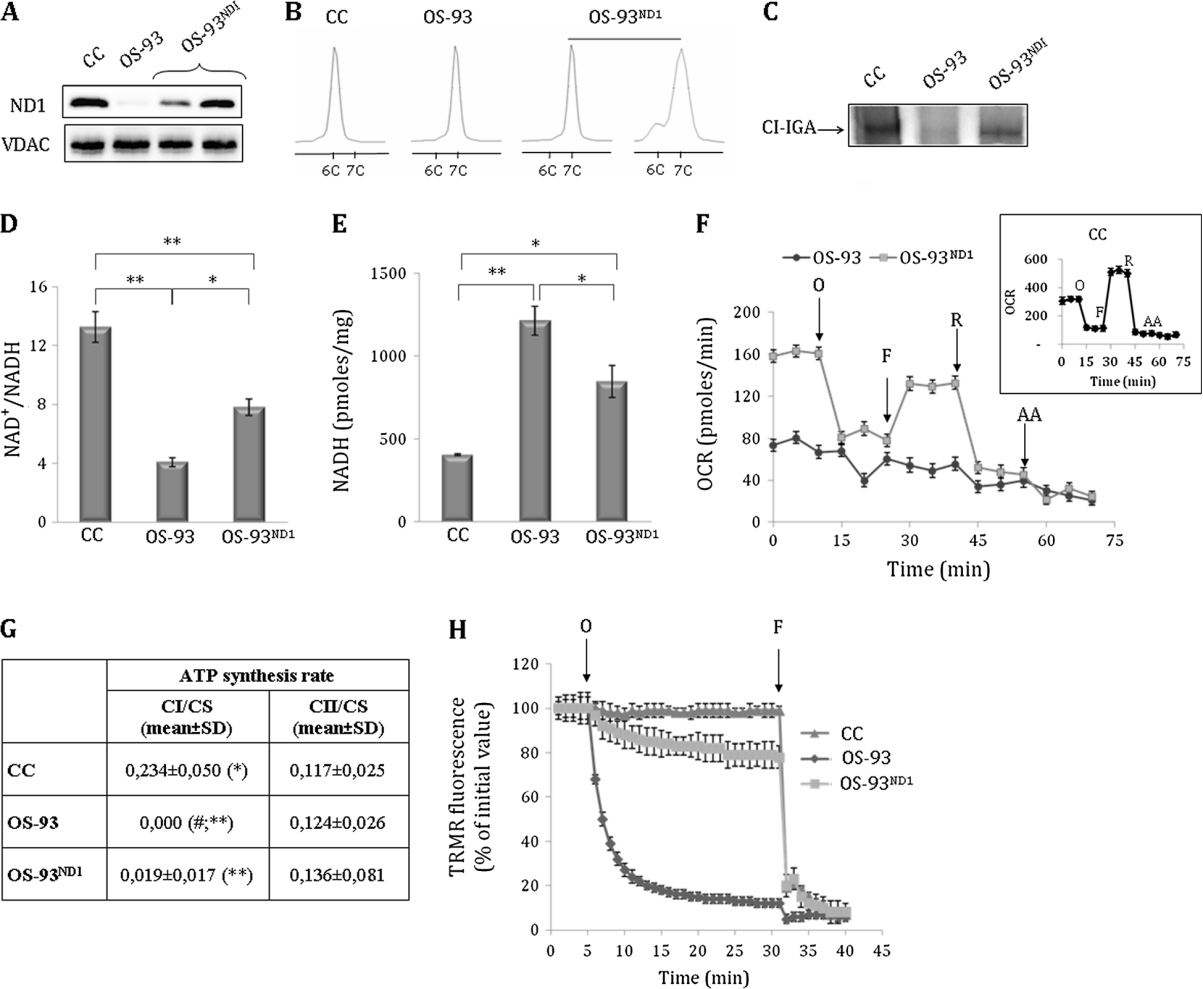
**Expression and functional effects of allotopic *nND1.*** (**A**) Western blot analysis for ND1 in control (CC), OS-93 and OS-93^ND1^ representative clones. Voltage-dependent anion channel (VDAC) was used as a loading control. One representative experiment of three is shown. (**B**) m.3571insC mutation load evaluation by fluorescent (F)-PCR. Wild-type and mutant fragments are distinguished based on the length of the homopolymeric stretch, where 7C corresponds to the C insertion. (**C**) Complex I in-gel activity (CI-IGA) assay in isolated mitochondria. CI-IGA band is indicated with an arrow. One representative experiment of four is shown**.** (**D**-**E**) NAD^+^/NADH ratio and NADH levels were measured in cell lysates. Data (mean ± SD) are expressed as pmoles of NADH and normalized for protein content (n = 3; ^*^P <0.05; **P <0.01). (**F**) Oxygen consumption rate (OCR). Measurements were performed upon injection of 1 μM oligomycin (O), 0.1 μM trifluorocarbonylcyanide phenylhydrazone (FCCP) (F), 1 μM rotenone (R) and 1 μM antimycin A (AA). The inset shows the OCR control cell line (CC) profile, analogous to that of OS-93^ND1^ cells. Data (mean ± standard error of the mean (SEM)) are expressed as pmoles of O_2_ per minute per 3 × 10^4^ cells (n = 3). (**G**) Mitochondrial ATP synthesis driven by pyruvate/malate and succinate, CI and CII substrates, respectively. CS, citrate synthase. (n = 4; ^#^undetectable value; ^**^OS-93 v*s* CC, *P* <0.01; ^**^OS-93^ND1^ vs OS-93, *P* <0.01; ^*^CC vs OS-93^ND1^, *P* <0.05). (**H**) Mitochondrial membrane potential evaluation in CC, OS-93 and OS-93^ND1^ cells. Arrows indicate the addition of 6 μM oligomycin (O) and 4 μM FCCP (F). Data are mean ± SEM (n = 6). Fluorescence readings following the addition of oligomycin and preceding that of FCCP revealed a statistically significant difference (*P* <0.05) for all time points between OS-93 and both CC and OS-93^ND1^ cells.

In order to recover CI function, we took advantage of the previously characterized osteosarcoma cell models (OS-93) bearing the quasi-homoplasmic disruptive m.3571insC mutation in the *MT-ND1* gene, which encodes the NADH dehydrogenase subunit 1 (ND1) of CI [14,15] (Additional file 5: Figure S3A). The mutation was complemented by recoding *MT-ND1* for cytosolic translation (*nND1*) using *in vitro* site-directed mutagenesis (Additional file 5: Figure S3B). A eukaryotic expression construct was designed with the aim to facilitate *nND1* mRNA targeting to the outer mitochondrial membrane [23] (Additional file 5: Figure S3C). ND1-null OS-93 cells were then transfected with the allotopic construct to generate OS-93^ND1^ clones, in which *nND1* mRNA expression was confirmed by qRT-PCR (Additional file 5: Figure S3D). Western blot analysis on enriched mitochondrial fractions showed that ND1 was present exclusively in OS-93^ND1^ allotopic clones (Figure 1A), indicating that the protein was correctly synthesized and localized within mitochondria. We next addressed the issue of genetic revertants by measuring the precise load of mutant mtDNA by F-PCR (Figure 1B) and DHPLC (Additional file 6: Figure S4). Genetic revertants were not carried along in subsequent analyses. Moreover, by sequencing the whole *MT-ND1* gene, clones were repeatedly controlled so that no additional mutations had accumulated that could complement the m.3571insC, for example, via recovery of the open reading frame. No difference in growth rate was found in glucose medium between CI-deficient and CI-competent cell clones (Additional file 7: Figure S5), indicating their basal metabolism to be mainly glycolytic, as it occurs generally when cells grow *in vitro* in presence of nutrients and oxygen. In order to verify whether the nND1 subunit was able to restore functional CI, the bioenergetics competence and redox state of cell clones were explored. CI-IGA analysis showed a band corresponding to fully assembled and functional CI in both wild-type mtDNA control (CC) and in OS-93^ND1^ cells, but not in OS-93 (Figure 1C), indicating a recovery of CI activity. This finding was confirmed by measuring the NAD^+^/NADH ratio, which was significantly recuperated by about 50% in OS-93^ND1^ compared to OS-93 cells (Figure 1D), likely due to recuperated consumption of NADH in the allotopic clone, albeit not as much as in control cells (Figure 1E). The basal respiration of OS-93^ND1^ clones was higher than in OS-93 and it was inhibited by oligomycin, indicating at least a partial rescue of phosphorylation capacity (Figure 1F). Furthermore, respiration was stimulated by addition of the uncoupler trifluorocarbonylcyanide phenylhydrazone (FCCP) and largely inhibited by rotenone and antimycin A (AA), confirming the rescue of CI function (Figure 1F). This finding was strengthened by the fact that OS-93^ND1^ cells partially but significantly recuperated CI-driven ATP synthesis compared to OS-93, as evaluated in digitonin-permeabilized cells in the presence of specific substrates (Figure 1G). No difference in CII-driven ATP synthesis was detected between allotopic and ND1-null cells, suggesting no alteration of the remaining spans of oxidative phosphorylation (Figure 1G). Moreover, similarly to CC, mitochondrial membrane potential (ΔΨ_m_) was maintained in allotopic cells after the addition of the ATP-synthase inhibitor oligomycin, whereas OS-93 cells rapidly depolarized (Figure 1H). Taken together, these results demonstrate that ND1 allotopic expression permitted recovery of a functional CI and rescued cellular bioenergetics competence.

### Complex I-competent allotopic clones recover tumorigenic potential

**Figure 2:**
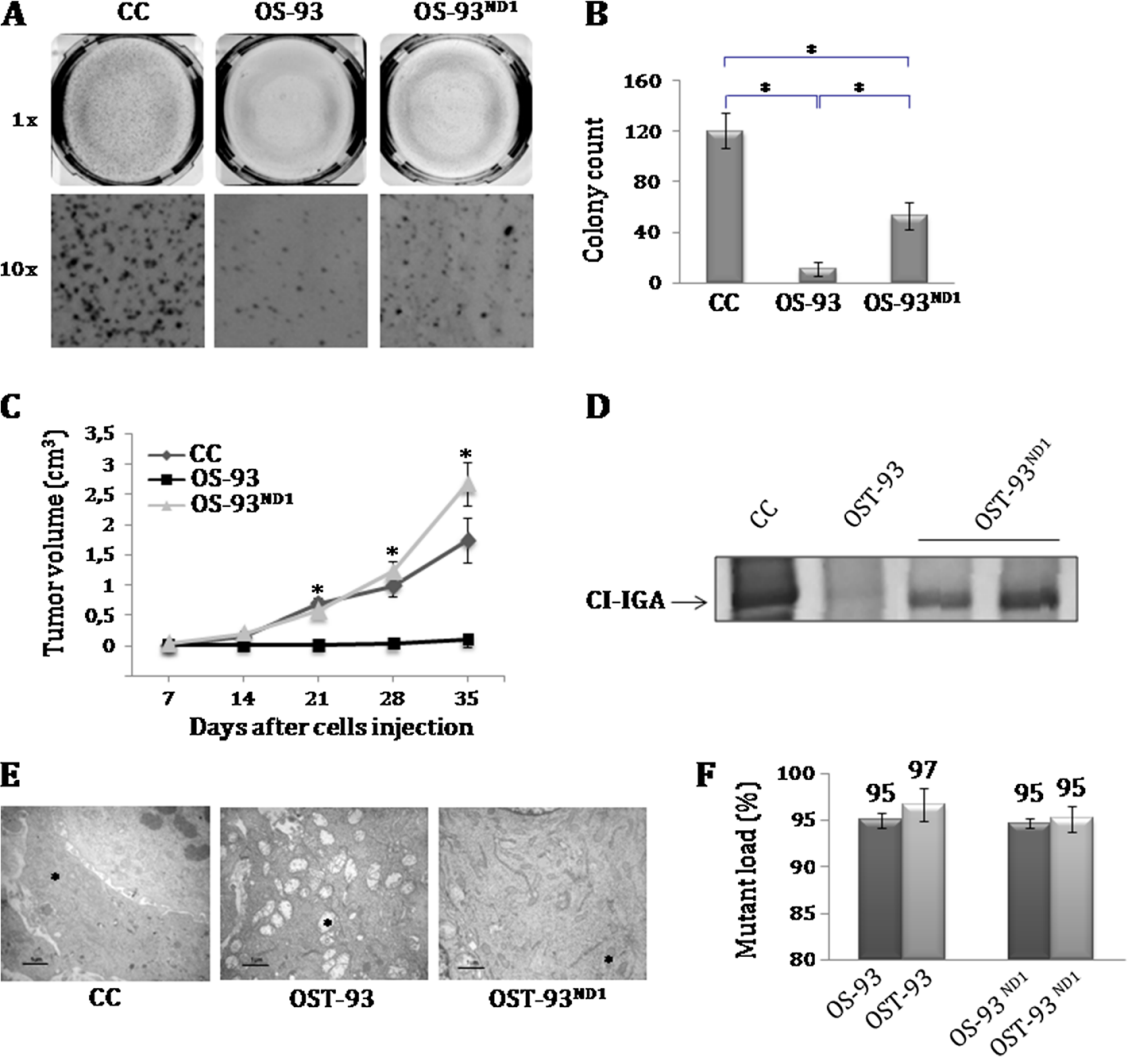
**Complex I (CI) function is required for recovery of tumorigenic potential *in vitro *and *in vivo.*** (**A**) Representative images of anchorage-independent colony growth in soft agar of control (CC), OS-93 and OS-93^ND1^ cell lines. (**B**) Colony count on soft agar plate after 7 days; data are mean ± SD (n = 3, ^*^P <0.05). (**C**) Tumor growth induced upon injection of CC, OS-93 and OS-93^ND1^ cell lines in nude mice. Data are mean ± standard error of the mean (SEM) (n = 3, 5 to 10 animals inoculated in each experiment; ^*^P <0.05; ^*^CC and OS-93^ND1^ versus OS-93). (**D**) CI-in gel activity (IGA) assay in tumor homogenates from CC, OST-93 and OST-93^ND1^ tumors. One representative experiment of three is shown. (**E**) Representative electron micrographs of CC, OST-93 and OST-93^ND1^ tumors. Asterisks indicate diverse mitochondrial morphology. (**F**) Fluorescent-PCR analysis of the m.3571insC in OS-93 and OS-93^ND1^ cell lines and xenografts (OST). All xenografts maintained the same m.3571insC mutant load as their corresponding cell lines (>90%).

To address the question of whether CI function is required for cancer cell growth, CC, OS-93 and OS-93^ND1^ were tested for their capacity to grow in an anchorage-independent manner. Allotopically complemented cells formed larger and significantly more numerous colonies than their mock clones (Figure 2A-B). Cells were injected in nude mice to determine their tumorigenic potential *in vivo*. Similar to controls, OS-93^ND1^-derived tumors (OST-93^ND1^) grew significantly larger than those derived from OS-93 cells (OST-93) (Figure 2C), demonstrating that the recovery of CI function *in vivo* (Figure 2D) is required for tumor growth. We have previously demonstrated that in the presence of a quasi-homoplasmic m.3571insC mutation, mitochondrial morphology is heavily deranged [15]. In fact, electron micrographs of OST-93 tumors showed large mitochondria with clear matrix and almost total loss of cristae (Figure 2E). On the other hand, OST-93^ND1^ and CC tumors presented with a population of mitochondria mostly with darker matrix and normal cristae (Figure 2E), indicating that the recovery of CI function was strictly associated with a recuperation of a normal mitochondrial morphology. These findings confirm the beneficial effects of allotopic ND1 expression on mitochondria *in vivo*. In order to rule out that such recovery might be due to a genetic reversion occurring during xenograft growth, we resequenced the mtDNA derived from the tumors and no other mutations apart from the m.3571insC were detected. Occurrence of revertant genotypes was also excluded by F-PCR (Figure 2F) and DHPLC analysis (Additional file 8: Figure S6). Overall, these data clearly indicate that CI function is required to sustain tumor growth *in vivo*.

We next addressed the involvement of ROS, since they have been shown to positively contribute to tumor growth and metastasis [18,51]. Hydrogen peroxide and superoxide levels were measured in the absence and in presence of AA (Additional file 4; Methods), an inhibitor of complex III (CIII) and the main superoxide inducer [17]. We previously reported that CI-ablated cells may be expected to produce fewer radicals, due to lack of one of the two ROS production sites [14,15], a trend we also observed here between CC and both OS-93 and allotopic OS-93^ND1^ (Additional file 9: Figure S7A-B), albeit not significant. Further, upon AA treatment, a significant increase in ROS levels was observed only in CC, whereas no increase was shown to occur in OS-93 and OS-93^ND1^ cells. These findings suggest that the severe CI mutation may not permit the normal electron flow through the complex and the rest of the respiratory chain, failing to ensure a minimal amount of electrons required for production of radicals, even in the presence of inhibited CIII. In allotopic cells, we failed to detect a rescue of ROS levels, indicating that the amount of fully re-assembled CI was lower than in CC cells, finally allowing us to rule out a major ROS contribution in determining the different tumorigenic potential of allotopic compared to CI-deficient cells.

### Global transcriptomic profiling reveals a HIF-1α-regulated Warburg phenotype in allotopic tumors

**Figure 3:**
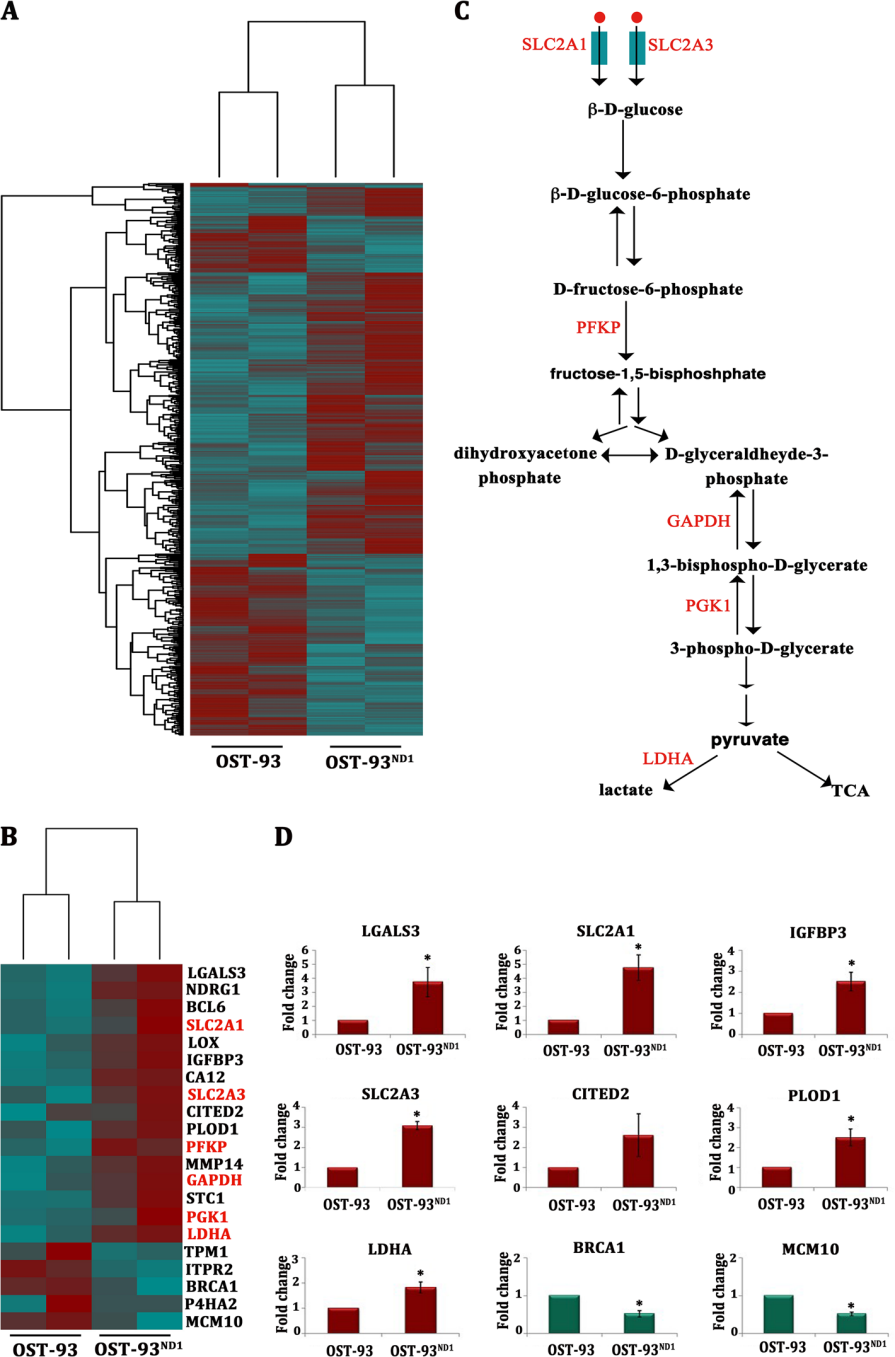
**Transcriptional profile of OST-93 and OST-93**^**ND1 **^**xenografts.** (**A**) Heatmap displaying expression levels of the 521 differentially expressed (DE) genes in the samples analyzed. Dark red = upregulated genes; cyan = downregulated genes. **(B)** The transcriptional profile of the Warburg phenotype**.** Heatmap showing gene expression profile of 21 DE genes from the hypoxia inducible factor-1α (HIF-1α) activation pathway. Genes are ordered by decreasing log_2_ fold change. Glucose transporters and glycolytic genes are marked in red. **(C)** Glucose uptake and glycolytic reactions. Genes overexpressed in OST-93^ND1^ xenografts are labeled in red. Red circles represent glucose molecules. **(D)** Quantitative real-time PCR validation performed on biological replicates of OST-93 (n = 2) and OST-93^ND1^ (n = 7) for 9/21 HIF-1α-responsive genes found DE in RNA-Seq (^*^P <0.05).

With the aim of dissecting the molecular pathways determining tumor growth or arrest in dependence of CI recovery, we next conducted a global transcriptomic profiling on OST-93 and OST-93^ND1^ xenografts. We found 521 DE genes, out of which 226 upregulated in OST-93 and 296 in OST-93^ND1^ samples (Figure 3A and Additional file 2: Table S1), with fold changes ranging between 2.0 and 13.8. The most significant GO categories among OST-93^ND1^ upregulated genes included the activation of the translational apparatus (Additional file 10: Table S2).

We have previously demonstrated that CI-deficient tumors are unable to respond to hypoxia via the destabilization of transcription factor HIF-1α [14,15]. In order to assess the involvement of HIF-1α-responsive targets, we specifically looked at such pathways within the set of DE genes. Interestingly, 21 out of 521 genes were downstream targets of HIF-1α (Figure 3B), most of which were known to be overexpressed during the hypoxia response. Remarkably, HIF-1α-responsive *NDRG1*, *LGALS3* and *IGFBP3*, overexpressed in OST-93^ND1^ tumors, were among the top-ranked DE genes detected here, with a false discovery rate (FDR) <5% (Additional file 2: Table S1). In agreement with data on known HIF-1α-repressed gene targets, a significant under-expression of *MCM10*, *BRCA1* and *TPM1* was found in OST-93^ND1^ tumors, suggesting an overall activation of the HIF-1α-regulated pathway occurring exclusively in allotopic xenografts (Figure 3B). Among HIF-1α-responsive genes, a cluster of four belonging to the glycolytic pathway (*PFKP*, *GAPDH*, *PGK1* and *LDHA*) and two encoding glucose transporters (*SLC2A1*, *SLC2A3*) were significantly overexpressed in OST-93^ND1^ tumors (Figure 3B-C). No other significant differential expression was evident from the transcriptomic data analysis regarding genes involved in cellular metabolism, indicating that glycolysis may be prevalently responsible for the higher growth ability of CI-competent cancer cells, suggesting the existence of a Warburg transcriptional profile in such tumors. Transcriptomic data were confirmed and validated by qRT-PCR (Figure 3D), which highly correlated (*R*^2^ = 0.91) with the RNA-Seq data (Additional file 11: Figure S8).

### HIF-1α stabilization occurs upon CI recovery and decreased α-KG/SA ratio

**Figure 4:**
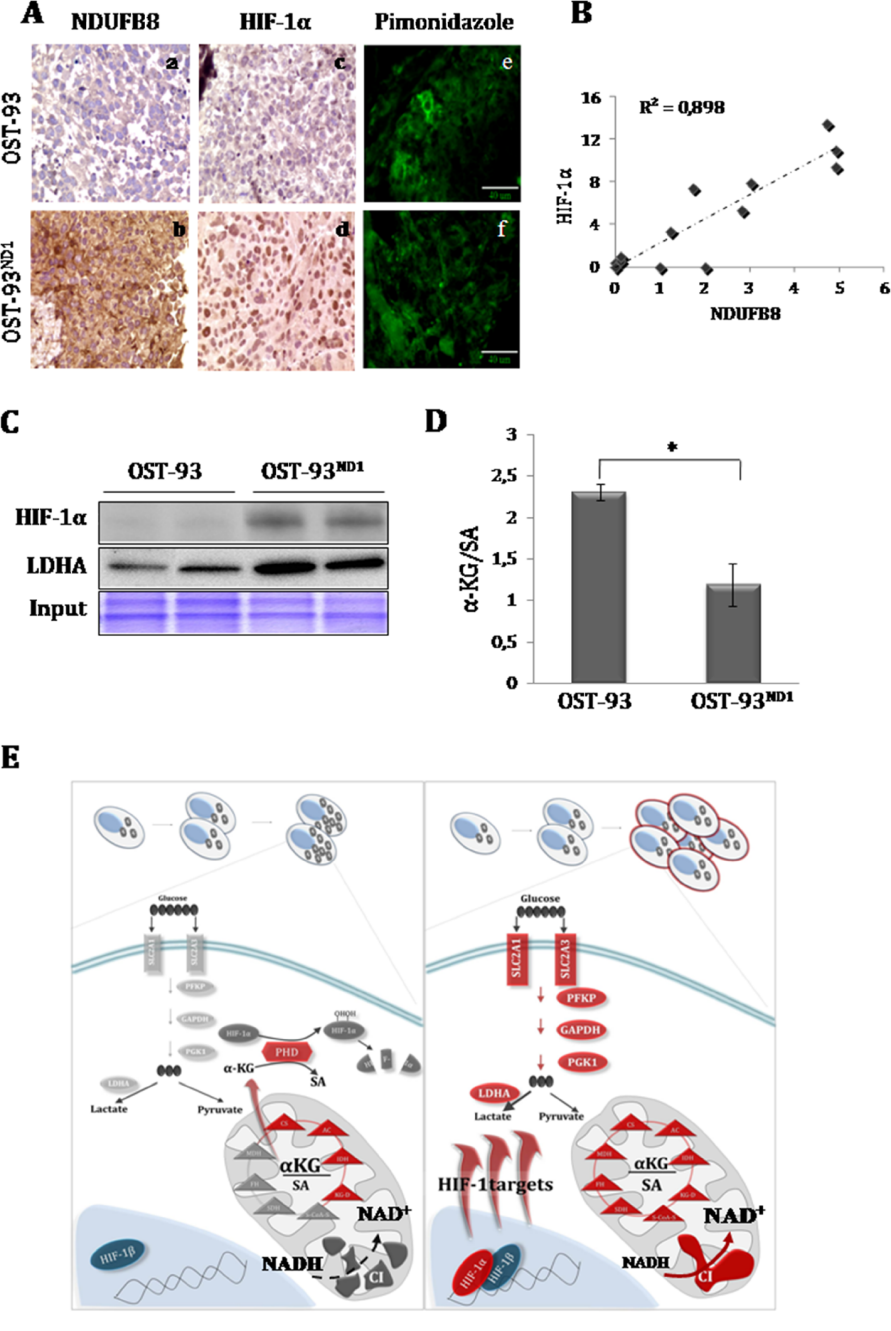
**Complex I (CI) rescue correlates with decrease in α-ketoglutarate (α-KG)/ succinate (SA) ratio and recovery of hypoxia inducible factor-1α (HIF-1α) stabilization.** (**A**) Representative immunohistochemical (IHC) analysis of CI (NDUFB8) and HIF-1α in OST-93 and OST-93^ND1^ xenografts. Positive NDUFB8 (b) and HIF-1α staining (d) is observed in OST-93^ND1^ but not in OST-93 xenografts (a, c); magnification 100×. Pimonidazole staining of representative OST-93 and OST-93^ND1^ xenografts (e, f); magnification 63×. (**B**) Correlation between NDUFB8 and HIF-1α IHC staining scores. X-axis represents NDUFB8 staining scores obtained considering the percentage of positive cells and staining intensity. Y-axis represents the percentage of HIF-1α-positive nuclei. **(C)** Western blot analysis of HIF-1α and lactate dehydrogenase A (LDHA) protein levels in OST-93 and OST-93^ND1^ tumors. Coomassie staining was used as loading control (input). (**D**) The ratio of α-KG and SA levels was calculated by measurements for each metabolite in OST-93- and OST-93^ND1^-derived cell lines. Data are mean ± SD (n = 3, ^*^P <0.05). (**E**) Scheme of the metabolic changes in the absence/recovery of functional CI in cancer cells. Non-functional CI leading to an increase of the α-KG/SA ratio (left panel), which may foster activity of prolyl-hydroxylases (PHDs) with subsequent HIF-1α degradation even at low oxygen. Inactivation of HIF-1α leads to the downregulation of glycolysis needed to compensate for the defective mitochondrial respiration. This scenario may not allow the metabolic adaptation of tumor cells, possibly inducing a short-circuited mitochondrial compensatory proliferation. Due to a recuperated NADH consumption, CI rescue restores the α-KG/SA balance (right panel), hence not preventing HIF-1α stabilization. HIF-1α may therefore translocate into the nucleus with HIF-1β and activate transcription of target genes (red ovals and rectangles), among which those contributing to increase the glycolytic flux, hence conferring a Warburg phenotype and allowing tumor adaptation and growth. Red elements indicate activation or overexpression.

To assess whether restoring CI via *nND1* expression affected HIF-1α stabilization, IHC analysis was performed on OST-93 and OST-93^ND1^ xenografts. Positive staining of both the NDUFB8 CI subunit and HIF-1α was found only in OST-93^ND1^ tumors (Figure 4A panels a-d). Both OST-93 and OST-93^ND1^ masses positively stained with hypoxic marker pimonidazole, indicating that HIF-1α was not stabilized in OST-93 tumors despite the low-oxygen tension microenvironment *in vivo* (Figure 4A panels e-f). The strong association between CI and HIF-1α stabilization was furthermore evident from the high correlation (*R*^2^ = 0.898) of the NDUFB8 and HIF-1α IHC staining (Figure 4B). Moreover, the protein expression levels of HIF-1α and its downstream target *LDHA*, were increased only in OST-93^ND1^ xenografts, demonstrating that HIF-1α was only functional in CI-competent tumors (Figure 4C). Since HIF-1α turnover is known to be affected by the α-KG/SA ratio, we investigated the levels of these two tricarboxylic acid (TCA) cycle metabolites in OST-93- and OST-93^ND1^-derived cells. The α-KG/SA ratio was significantly higher in OST-93 compared to OST-93^ND1^ (Figure 4D), clearly indicating that CI function influenced the balance of these TCA cycle metabolites and in turn, permitted HIF-1α stabilization. Last, we attempted to verify whether HIF-1α was responsible for the augmented tumorigenic potential of allotopic compared to CI-deficient cells. To this aim, we used the potent HIF-1α stabilizer DMOG, a specific inhibitor of PHDs, to force HIF-1α stabilization in cells, and evaluated their tumorigenic potential in soft agar. Interestingly, DMOG-treated OS-93 CI-deficient cells displayed a significantly increased colony-forming ability by about 50% (*P* <0.05).

## Discussion

In the present study, we demonstrate that functional mitochondrial CI is required for the induction of the Warburg phenotype, namely the metabolic reprogramming of cancer cells towards glycolysis during tumor progression *in vivo*. Our results strongly indicate that the mechanism through which CI regulates the glycolytic shift is mediated by HIF-1α stabilization, allowing tumor cells to adapt to hypoxia and persist in proliferation.

In the last few years the Warburg effect has been widely debated, in particular with respect to the role of mitochondria in the regulation of cancer metabolism. In fact, at least three of the four potential causes of the Warburg effect revolve around metabolic processes that converge directly or indirectly to mitochondria. In this framework, it is interesting that two key enzymes of the TCA cycle, namely FH and SDH (complex II of the respiratory chain) are *de facto* tumor suppressor genes whose loss-of-function mutations provide a permissive environment for oncogenic hits to trigger transformation [52]. The widely shown mechanism through which they facilitate tumor growth/adaptation is the stabilization of HIF-1α, the master mediator of progression towards malignancy, via the imbalance of TCA cycle metabolites α-KG and SA [53]. Our results show that, in contrast to what occurs with FH and SDH, severe CI loss-of-function mutations impinge on the cells ability to stabilize HIF-1α despite hypoxia, via a shift in the α-KG/SA ratio in favor of α-KG. We attempted to provide a functional connection between these phenomena via the evaluation of the NAD^+^/NADH ratio, which we confirmed to be decreased in CI-deficient cells, likely due to a lower NADH consumption in absence of the complex. It has long been known that NADH accumulation contributes to the allosteric inhibition of α-KG dehydrogenase, with subsequent α-KG accumulation [54], which may lead to HIF-1α destabilization and hence triggering of pseudonormoxia.

These findings suggest that a novel two-way relationship exists between HIF-1α and mitochondria, besides the well-known negative regulation of pyruvate dehydrogenase by pyruvate dehydrogenase kinase 1, which is a known positive target of HIF-1α [12,13]. It has been proposed that a slowdown in oxidative metabolism may confer an advantage to tumor cells by decreasing their oxygen requirements and by inducing anabolic reactions [8,55]. Nevertheless, there is also evidence for a correlation between CI inhibition and HIF-1α destabilization, as shown using pharmacological treatment with respiratory chain inhibitors [56,57]. Moreover, several studies have shown that loss of respiratory CIII may contribute to ROS-mediated destabilization of HIF-1α [58,59], similar to the disassembling CI mutations that we have extensively reported [14,15]. We previously proved that the homoplasmic m.3571insC/*MT-ND1* mtDNA mutation causes a complete loss of CI activity, hampering tumor growth both *in vitro* and *in vivo*[15]. Such types of homoplasmic disruptive mutations impinging on CI function do occur in human cancers, where they are generally associated to the oncocytic phenotype and to an indolent, low aggressive behavior, and even to a higher chromosomal stability [14,21,60-63].

In order to provide a proof of concept that CI function is necessary for recovery of tumor growth, we complemented the OS-93 cell line with the wild-type *MT-ND1* using an allotopic expression strategy. The xenografts subjected to the next generation RNA-Seq technique showed significant upregulation of ribosomal biogenesis and protein translation in tumors with a functional CI (Additional file 10: Table S2), a profile previously defined as a molecular marker of cancer phenotype. In fact, an augmented synthesis of ribosomes, and consequently of proteins, can sustain the high rate of proliferation required by cancer cells [64,65]. Moreover, a significant upregulation of HIF-1α-responsive genes was detected in allotopic tumors, in particular those involved in glycolytic metabolism. They were all found to be overexpressed in cells with intact CI, defining a glycolytic transcriptional profile typical of fast-growing tumors [5,6,44,66]. The indication that such a HIF-1α-mediated Warburg profile is essential to tumor progression was provided by the rescue of anchorage-independent cell growth upon forced stabilization of the transcription factor by DMOG.

Further studies *in vivo* are warranted to definitely prove that pseudonormoxia is the main determinant of the decreased tumorigenic potential of CI-deficient cells. Nonetheless, our *in vitro* data support the hypothesis that a strong metabolic impairment impacts on the HIF-1α-dependent adaptive ability of cancer cells, likely via a TCA imbalance. In fact, HIF-1α degradation is mediated by the PHD enzymes, which hydroxylate the transcription factor using α-KG in a reaction that in turn produces SA, an allosteric inhibitor of the PHDs [53]. Recovery of CI activity was shown here to restore a physiological α-KG/SA ratio, indicating that TCA cycle reactivation may be necessary to provide the metabolic conditions for the induction of a hypoxic response with a consequent HIF-1α stabilization (Figure 4E). Such a condition may well depend on a re-balanced ratio of NAD^+^/NADH, which followed CI recovery. In fact, such a metabolite-sensing mechanism and allosteric enzyme regulation may represent a quick route through which cancer cells can respond to the selective pressure of the ever-changing tumor microenvironment, as several studies underline [67]. A similar regulation may be crucial to carry out the switch between glycolytic and oxidative metabolism in specific phases of tumor progression, as explained in a recently proposed wave-like model of regulation of tumor metabolism [6].

Revitalization of mitochondrial function has been proposed as one of the phases following hypoxic adaptation, when nutrients and oxygen are again available to cancer cells. In this context, inactivating CI mtDNA mutations, such as m.3571insC/*MT-ND1*, may exhibit their effect only at mitochondrial-dependent stages of tumor progression. These effects have been shown to vary, depending on mutant load and mutation type [21]. For instance, the m.3571insC mutation investigated here has been shown to exhibit anti-tumorigenic effects when present above the 83% threshold [15]. On the other hand, some missense CI mtDNA mutations have been demonstrated to promote tumor growth [18,19]. Overall, it is reasonable to suggest that although a certain degree of mitochondrial dysfunction can be advantageous when selective pressures operate, total impairment of OXPHOS may not sustain tumor progression. It is therefore of paramount importance to note that both the degree of heteroplasmy and the type of mutation (missense vs truncating) must be carefully taken into account when considering the prognostic value of such genetic markers, since they may determine a completely opposite tumor fate, according to whether CI is functional or not [15,20,21,68,69]. From our results, in fact, it has emerged that intact CI is needed for adaptation to hypoxia, which is in agreement with previous reports showing that HIF-1α stabilization requires functional OXPHOS in conditions of oxygen shortage [59,70].

## Conclusions

The role of CI in the induction of the Warburg phenotype and hypoxia response in cancer cells suggests that it may no longer be considered an enzyme that merely contributes to mitochondrial function, but that it may be pivotal in determining the fate of cancer cells after deregulation of their metabolic switch during tumor progression. Therefore, CI, far from being considered a tumor suppressor, ought more likely to be looked upon as a lethality modifier of cancer cells when complete loss-of-function mutations occur. Such concepts, epitomized by the definition of oncojanus mitochondrial mutations [15], has potential applications in establishing prognostic criteria, since genetic markers such as CI-disruptive mutations may have a strong impact on the successful metabolic adaptation of the neoplasia.

## Abbreviations

AA: Antimycin A; AC: Aconitase; α-KG: α-ketoglutarate; ANOVA: Analysis of variance; BN-PAGE: Blue native polyacrlylammide gel electrophoresis; bp: Base pair; CC: Control cell line; CI: Complex I; CS: Citrate synthase; DE: Differentially expressed; DHPLC: Denaturing high performance liquid chromatography; DMEM: Dulbecco’s modified Eagle’s medium; DMOG: Dimethyloxallylglycine; FBS: Fetal bovine serum; FCCP: Trifluorocarbonylcyanide phenylhydrazone; FDR: False discovery rate; FH: Fumarate hydratase; F-PCR: Fluorescent polymerase chain reaction; GO: Gene ontology; HIF-1α: Hypoxia inducible factor-1α; IDH: Isocitrate dehydrogenase; IGA: In gel activity; IHC: Immunohistochemical; IRS: Immunoreactivity score; KEGG: Kyoto encyclopedia of genes and genomes; KG-D: α-ketoglutarate dehydrogenase; LDHA,: Lactate dehydrogenase A; MDH: Malate dehydrogenase; mtDNA: Mitochondrial DNA; NGS-Trex: Next generation sequencing-TRanscriptome profile explorer; OCR: Oxygen consumption rate; OXPHOS: Oxidative phosphorylation; PBS: Phosphate-buffered saline; PHD: Prolyl-hydroxylase; qRT-PCR: Quantitative real-time PCR; rCRS: Revised Cambridge Reference Sequence; ROS: Reactive oxygen species; SA: Succinate; S-CoA-s: Succinyl-CoA synthase; SDH: Succinate dehydrogenase; TCA: Tricarboxylic acid; TMRM: Tetramethylrhodamine methyl esther; UTR: Untranslated region; VDAC: Voltage-dependent anion channel.

## Competing interests

The authors declare that they have no competing interests.

## Authors’ contributions

CC^1^ and IK performed transcriptomic data analyses and validation and wrote the paper; LI performed allotopic complementation and biochemical measurements and wrote the paper; MAC performed respiratory complexes assembly assays; CB, RF performed NAD^+^ and NADH measurements; MC^2^ performed soft-agar assays; PLL, PN, GN, CDG performed *in vivo* experiments; AG, MR and VC provided counseling for biochemical studies; VG performed and analyzed SeaHorse experiments; MC^6^, FM, CM, AT performed RNA-Seq experiments; CMB performed electron microscopy and corrected the paper; CC^9^ performed immunohistochemical experiments; MA supervised bioinformatics analyses; GR provided counseling and funding for molecular genetics experiments; GG and AMP designed and supervised research, analyzed data and wrote the paper. All authors read and approved the final manuscript.

## Authors’ information

Co-first authors: Claudia Calabrese, Luisa Iommarini and Ivana Kurelac; co-last authors: Giuseppe Gasparre and Anna Maria Porcelli.

## Supplementary Material

Additional file 1: Figure S1 DMOG effects validation. Western blot analysis for hypoxia inducible factor-1α (HIF-1α) protein on cell lysates upon treatment with 1 μM dimethyloxallylglycine (DMOG) indicate presence of stabilized HIF-1α only in the treated cells despite normoxic culture conditions. Tubulin was used as a loading control. Click here for file

Additional file 2: Table S1 Raw sequencing data together with the 521 genes differentially expressed among the two groups of samples (OST-93, OST-93^ND1^). Click here for file

Additional file 3: Figure S2 FastQC analysis of fastq sequences from the four data sets of samples. Panel A and B refer respectively to OST-93 samples and to OST-93^ND1^ samples. **(a-c)** the distribution of the average quality scores per sequence within the set always ranges between 30 to 39 QS (quality score) values; **(b-d)** QS distribution per base position across the maximum read length observed within the data set. In all four samples the lower quartile associated to the 300^th^ position of the read (average read length for each sequences data set) is always above 20. Click here for file

Additional file 4 Additional information. Click here for file

Additional file 5: Figure S3 Generation of allotopic *nND1*. **(A)** Elecropherogram showing m.3571insC in OS-93 cell line. **(B)** Amino acid sequence of ND1. The sites of directed mutagenesis performed to optimize codon usage for cytosolic translation of methionine (M) and tryptophan (W) are underlined and indicated in bold. **(C)** Scheme of the allotopic expression vector containing the *nND1* transgene with the 3^′^- and 5^′^-UTR from the nuclear-encoded COX10 mitochondrial protein. Antibiotic resistance genes, CMV promoter, SV40 and f1 origin of replication are also indicated. **(D)** qRT-PCR showing mRNA expression of the cytosolically expressed *nND1* construct in OS-93 cells. Click here for file

Additional file 6: Figure S4 Revertans exclusion. Denaturing high performance liquid chromatography (DHPLC) analysis of the allotopically complemented OS-93 cells. OS-93^ND1#1^ (cyan) has maintained the same m.3571insC mutant load as the original OS-93 cell line (red), while OS-93^ND1#2^ is a representative example of a revertant clone in which a higher proportion of wild-type molecules is evident from the elevated elution peak (pink). Click here for file

Additional file 7: Figure S5 Cellular growth determination. Cellular growth in DMEM-high glucose. Data represent mean ± standard error of the mean (n = 3). Click here for file

Additional file 8: Figure S6 Denaturing high performance liquid chromatography (DHPLC) analysis of OS-93 and OS-93^ND1^ clones and corresponding xenografts. Elution curves indicate maintenance of the m.3571insC genotype (mutant loads >90%). Click here for file

Additional file 9: Figure S7 Evaluation of reactive oxygen species (ROS) levels. **(A)** Hydrogen peroxide levels were measured using 2 μM H_2_DCFDA in the absence (basal) and presence of 1 uM antimycin A (AA) for 1 h, as described in Additional file 4; Methods. Data (mean ± SD, n = 3; ^*^P <0.05) are expressed as ratio of fluorescence of H_2_DCFDA and calcein-AM. **(B)** Superoxide production was determined using 5 μM MitoSOX-Red, as described in Additional file 4; Methods. Images are representative of three different experiments. Magnification 63×/1.4. Ten images were acquired for each experiment. Click here for file

Additional file 10: Table S2 Gene Ontology (GO) and Kyoto Encyclopedia of Genes and Genomes (KEGG) categories enrichment results: 34 GO categories and 8 KEGG categories were found significantly enriched among all the 521 differentially expressed genes. GO enrichments were also found by the GeneMania software among the two sets of only overexpressed genes per sample (226 upregulated genes within the OST-93 group and 296 upregulated genes within the OST-93^ND1^ group). Click here for file

Additional file 11: Figure S8 Correlation between RNA-Seq analysis and qRT-PCR performed for 15/521 DE genes. Pearsons correlation coefficient was calculated using log_2_fold values. Click here for file

## References

[B1] Koppenol WH Bounds PL Dang CV Otto Warburg’s contributions to current concepts of cancer metabolism Nat Rev Cancer 2010 11 325 337 21508971 10.1038/nrc3038

[B2] Warburg O Posener K Negelein E Uber den Stoffwechsel der Carcinomzelle Biochem Zeitschr 1924 152 309 344

[B3] Ward PS Thompson CB Metabolic reprogramming: a cancer hallmark even Warburg did not anticipate Cancer Cell 2012 21 297 308 10.1016/j.ccr.2012.02.014 22439925 PMC3311998

[B4] DeBerardinis RJ Lum JJ Hatzivassiliou G Thompson CB The biology of cancer: metabolic reprogramming fuels cell growth and proliferation Cell Metab 2008 7 11 20 10.1016/j.cmet.2007.10.002 18177721

[B5] Moreno-Sanchez R Rodriguez-Enriquez S Marin-Hernandez A Saavedra E Energy metabolism in tumor cells Febs J 2007 274 1393 1418 10.1111/j.1742-4658.2007.05686.x 17302740

[B6] Smolkova K Plecita-Hlavata L Bellance N Benard G Rossignol R Jezek P Waves of gene regulation suppress and then restore oxidative phosphorylation in cancer cells Int J Biochem Cell Biol 2010 43 950 968 20460169 10.1016/j.biocel.2010.05.003

[B7] Chandra D Singh KK Genetic insights into OXPHOS defect and its role in cancer Biochim Biophys Acta 1807 2011 620 625 10.1016/j.bbabio.2010.10.023 PMC4681500 21074512

[B8] Galluzzi L Morselli E Kepp O Vitale I Rigoni A Vacchelli E Michaud M Zischka H Castedo M Kroemer G Mitochondrial gateways to cancer Mol Aspects Med 2010 31 1 20 10.1016/j.mam.2009.08.002 19698742

[B9] Chavez A Miranda LF Pichiule P Chavez JC Mitochondria and hypoxia-induced gene expression mediated by hypoxia-inducible factors Ann NY Acad Sci 2008 1147 312 320 10.1196/annals.1427.021 19076453

[B10] Semenza GL Hypoxia-inducible factor 1 (HIF-1) pathway Sci STKE 2007 2007 cm8 10.1126/stke.4072007cm8 17925579

[B11] Semenza GL Roth PH Fang HM Wang GL Transcriptional regulation of genes encoding glycolytic enzymes by hypoxia-inducible factor 1 J Biol Chem 1994 269 23757 23763 8089148

[B12] Kim JW Tchernyshyov I Semenza GL Dang CV HIF-1-mediated expression of pyruvate dehydrogenase kinase: a metabolic switch required for cellular adaptation to hypoxia Cell Metab 2006 3 177 185 10.1016/j.cmet.2006.02.002 16517405

[B13] Papandreou I Cairns RA Fontana L Lim AL Denko NC HIF-1 mediates adaptation to hypoxia by actively downregulating mitochondrial oxygen consumption Cell Metab 2006 3 187 197 10.1016/j.cmet.2006.01.012 16517406

[B14] Porcelli AM Ghelli A Ceccarelli C Lang M Cenacchi G Capristo M Pennisi LF Morra I Ciccarelli E Melcarne A Bartoletti-Stella A Salfi N Tallini G Martinuzzi A Carelli V Attimonelli M Rugolo M Romeo G Gasparre G The genetic and metabolic signature of oncocytic transformation implicates HIF1alpha destabilization Hum Mol Genet 2010 19 1019 1032 10.1093/hmg/ddp566 20028790

[B15] Gasparre G Kurelac I Capristo M Iommarini L Ghelli A Ceccarelli C Nicoletti G Nanni P De Giovanni C Scotlandi K Betts CM Carelli V Lollini PL Romeo G Rugolo M Porcelli AM A mutation threshold distinguishes the antitumorigenic effects of the mitochondrial gene MTND1, an oncojanus function Cancer Res 2011 71 6220 6229 10.1158/0008-5472.CAN-11-1042 21852384

[B16] Brandt U Energy converting NADH:quinone oxidoreductase (complex I) Annu Rev Biochem 2006 75 69 92 10.1146/annurev.biochem.75.103004.142539 16756485

[B17] Koopman WJ Nijtmans LG Dieteren CE Roestenberg P Valsecchi F Smeitink JA Willems PH Mammalian mitochondrial complex I: biogenesis, regulation, and reactive oxygen species generation Antioxid Redox Signal 2010 12 1431 1470 10.1089/ars.2009.2743 19803744

[B18] Ishikawa K Takenaga K Akimoto M Koshikawa N Yamaguchi A Imanishi H Nakada K Honma Y Hayashi J ROS-generating mitochondrial DNA mutations can regulate tumor cell metastasis Science 2008 320 661 664 10.1126/science.1156906 18388260

[B19] Sharma LK Fang H Liu J Vartak R Deng J Bai Y Mitochondrial respiratory complex I dysfunction promotes tumorigenesis through ROS alteration and AKT activation Hum Mol Genet 2011 20 4605 4616 10.1093/hmg/ddr395 21890492 PMC3209831

[B20] Park JS Sharma LK Li H Xiang R Holstein D Wu J Lechleiter J Naylor SL Deng JJ Lu J Bai Y A heteroplasmic, not homoplasmic, mitochondrial DNA mutation promotes tumorigenesis via alteration in reactive oxygen species generation and apoptosis Hum Mol Genet 2009 18 1578 1589 10.1093/hmg/ddp069 19208652 PMC2733816

[B21] Iommarini L Calvaruso MA Kurelac I Gasparre G Porcelli AM Complex I impairment in mitochondrial diseases and cancer: Parallel roads leading to different outcomes Int J Biochem Cell Biol 2013 45 1 47 63 10.1016/j.biocel.2012.05.016 22664328

[B22] Zielke A Tezelman S Jossart GH Wong M Siperstein AE Duh QY Clark OH Establishment of a highly differentiated thyroid cancer cell line of Hurthle cell origin Thyroid 1998 8 475 483 10.1089/thy.1998.8.475 9669284

[B23] Bonnet C Kaltimbacher V Ellouze S Augustin S Benit P Forster V Rustin P Sahel JA Corral-Debrinski M Allotopic mRNA localization to the mitochondrial surface rescues respiratory chain defects in fibroblasts harboring mitochondrial DNA mutations affecting complex I or v subunits Rejuvenation Res 2007 10 127 144 10.1089/rej.2006.0526 17518546

[B24] Rozen S Skaletsky H Primer3 on the WWW for general users and for biologist programmers Methods Mol Biol 2000 132 365 386 10547847 10.1385/1-59259-192-2:365

[B25] OligoAnalyzer [http://eu.idtdna.com/analyzer/Applications/OligoAnalyzer/]

[B26] Guerra F Kurelac I Cormio A Zuntini R Amato LB Ceccarelli C Santini D Cormio G Fracasso F Selvaggi L Resta L Attimonelli M Gadaleta MN Gasparre G Placing mitochondrial DNA mutations within the progression model of type I endometrial carcinoma Hum Mol Genet 2011 20 2394 2405 10.1093/hmg/ddr146 21470976

[B27] Kurelac I Lang M Zuntini R Calabrese C Simone D Vicario S Santamaria M Attimonelli M Romeo G Gasparre G Searching for a needle in the haystack: comparing six methods to evaluate heteroplasmy in difficult sequence context Biotechnol Adv 2011 30 363 371 21689740 10.1016/j.biotechadv.2011.06.001

[B28] Calvaruso MA Smeitink J Nijtmans L Electrophoresis techniques to investigate defects in oxidative phosphorylation Methods 2008 46 281 287 10.1016/j.ymeth.2008.09.023 18948205

[B29] Wittig I Karas M Schagger H High resolution clear native electrophoresis for in-gel functional assays and fluorescence studies of membrane protein complexes Mol Cell Proteomics 2007 6 1215 1225 10.1074/mcp.M700076-MCP200 17426019

[B30] Porcelli AM Angelin A Ghelli A Mariani E Martinuzzi A Carelli V Petronilli V Bernardi P Rugolo M Respiratory complex I dysfunction due to mitochondrial DNA mutations shifts the voltage threshold for opening of the permeability transition pore toward resting levels J Biol Chem 2009 284 2045 2052 19047048 10.1074/jbc.M807321200

[B31] Jones DP Determination of pyridine dinucleotides in cell extracts by high-performance liquid chromatography J Chromatogr 1981 225 446 449 10.1016/S0378-4347(00)80293-5 7298779

[B32] Giorgio V Petronilli V Ghelli A Carelli V Rugolo M Lenaz G Bernardi P The effects of idebenone on mitochondrial bioenergetics Biochim Biophys Acta 1817 2011 363 369 10.1016/j.bbabio.2011.10.012 PMC3265671 22086148

[B33] FastQC http://www.bioinformatics.babraham.ac.uk/projects/fastqc

[B34] NGS-Trex http://www.ngs-trex.org

[B35] Robinson MD McCarthy DJ Smyth GK edgeR: a Bioconductor package for differential expression analysis of digital gene expression data Bioinformatics 2010 26 139 140 10.1093/bioinformatics/btp616 19910308 PMC2796818

[B36] Bioconductor http://www.bioconductor.org

[B37] Young MD Wakefield MJ Smyth GK Oshlack A Gene ontology analysis for RNA-seq: accounting for selection bias Genome Biol 2010 11 R14 10.1186/gb-2010-11-2-r14 20132535 PMC2872874

[B38] Warde-Farley D Donaldson SL Comes O Zuberi K Badrawi R Chao P Franz M Grouios C Kazi F Lopes CT Maitland A Mostafavi S Montojo J Shao Q Wright G Bader GD Morris Q The GeneMANIA prediction server: biological network integration for gene prioritization and predicting gene function Nucleic Acids Res 2010 38 Web Server issue W214 220 20576703 10.1093/nar/gkq537 PMC2896186

[B39] Manalo DJ Rowan A Lavoie T Natarajan L Kelly BD Ye SQ Garcia JG Semenza GL Transcriptional regulation of vascular endothelial cell responses to hypoxia by HIF-1 Blood 2005 105 659 669 10.1182/blood-2004-07-2958 15374877

[B40] Greijer AE van der Groep P Kemming D Shvarts A Semenza GL Meijer GA van de Wiel MA Belien JA van Diest PJ van der Wall E Up-regulation of gene expression by hypoxia is mediated predominantly by hypoxia-inducible factor 1 (HIF-1) J Pathol 2005 206 291 304 10.1002/path.1778 15906272

[B41] Nickols NG Jacobs CS Farkas ME Dervan PB Modulating hypoxia-inducible transcription by disrupting the HIF-1-DNA interface ACS Chem Biol 2007 2 561 571 10.1021/cb700110z 17708671 PMC3060759

[B42] Benita Y Kikuchi H Smith AD Zhang MQ Chung DC Xavier RJ An integrative genomics approach identifies Hypoxia Inducible Factor-1 (HIF-1)-target genes that form the core response to hypoxia Nucleic Acids Res 2009 37 4587 4602 10.1093/nar/gkp425 19491311 PMC2724271

[B43] Wang M Li W Chang GQ Ye CS Ou JS Li XX Liu Y Cheang TY Huang XL Wang SM MicroRNA-21 regulates vascular smooth muscle cell function via targeting tropomyosin 1 in arteriosclerosis obliterans of lower extremities Arterioscler Thromb Vasc Biol 2011 31 2044 2053 10.1161/ATVBAHA.111.229559 21817107

[B44] Semenza GL Targeting HIF-1 for cancer therapy Nat Rev Cancer 2003 3 721 732 13130303 10.1038/nrc1187

[B45] entry H-Ag [http://www.ncbi.nlm.nih.gov/gene/3091]

[B46] R-project www.r-project.org

[B47] Bartoletti-Stella A Salfi NC Ceccarelli C Attimonelli M Romeo G Gasparre G Mitochondrial DNA mutations in oncocytic adnexal lacrimal glands of the conjunctiva Arch Ophthalmol 2011 129 664 666 21555623 10.1001/archophthalmol.2011.95

[B48] Remmele W Stegner HE: [Recommendation for uniform definition of an immunoreactive score (IRS) for immunohistochemical estrogen receptor detection (ER-ICA) in breast cancer tissue] Pathologe 1987 8 138 140 3303008

[B49] Ricciardiello L Ceccarelli C Angiolini G Pariali M Chieco P Paterini P Biasco G Martinelli GN Roda E Bazzoli F High thymidylate synthase expression in colorectal cancer with microsatellite instability: implications for chemotherapeutic strategies Clin Cancer Res 2005 11 4234 4240 10.1158/1078-0432.CCR-05-0141 15930362

[B50] Ceccarelli C Santini D Chieco P Lanciotti C Taffurelli M Paladini G Marrano D Quantitative p21(waf-1)/p53 immunohistochemical analysis defines groups of primary invasive breast carcinomas with different prognostic indicators Int J Cancer 2001 95 128 134 10.1002/1097-0215(20010320)95:2<128::AID-IJC1022>3.0.CO;2-D 11241324

[B51] Petros JA Baumann AK Ruiz-Pesini E Amin MB Sun CQ Hall J Lim S Issa MM Flanders WD Hosseini SH Marshall FF Wallace DC mtDNA mutations increase tumorigenicity in prostate cancer Proc Natl Acad Sci USA 2005 102 719 724 10.1073/pnas.0408894102 15647368 PMC545582

[B52] King A Selak MA Gottlieb E Succinate dehydrogenase and fumarate hydratase: linking mitochondrial dysfunction and cancer Oncogene 2006 25 4675 4682 10.1038/sj.onc.1209594 16892081

[B53] Selak MA Armour SM MacKenzie ED Boulahbel H Watson DG Mansfield KD Pan Y Simon MC Thompson CB Gottlieb E Succinate links TCA cycle dysfunction to oncogenesis by inhibiting HIF-alpha prolyl hydroxylase Cancer Cell 2005 7 77 85 10.1016/j.ccr.2004.11.022 15652751

[B54] Bunik VI Buneeva OA Gomazkova VS Change in alpha-ketoglutarate dehydrogenase cooperative properties due to dihydrolipoate and NADH FEBS Lett 1990 269 252 254 10.1016/0014-5793(90)81166-L 2117555

[B55] Jones RG Thompson CB Tumor suppressors and cell metabolism: a recipe for cancer growth Genes Dev 2009 23 537 548 10.1101/gad.1756509 19270154 PMC2763495

[B56] Agani FH Pichiule P Carlos Chavez J LaManna JC Inhibitors of mitochondrial complex I attenuate the accumulation of hypoxia-inducible factor-1 during hypoxia in Hep3B cells Comp Biochem Physiol A Mol Integr Physiol 2002 132 107 109 10.1016/S1095-6433(01)00535-9 12062197

[B57] Liu Y Morgan JB Coothankandaswamy V Liu R Jekabsons MB Mahdi F Nagle DG Zhou YD The Caulerpa pigment caulerpin inhibits HIF-1 activation and mitochondrial respiration J Nat Prod 2009 72 2104 2109 10.1021/np9005794 19921787 PMC2798910

[B58] Guzy RD Hoyos B Robin E Chen H Liu L Mansfield KD Simon MC Hammerling U Schumacker PT Mitochondrial complex III is required for hypoxia-induced ROS production and cellular oxygen sensing Cell Metab 2005 1 401 408 10.1016/j.cmet.2005.05.001 16054089

[B59] Mansfield KD Guzy RD Pan Y Young RM Cash TP Schumacker PT Simon MC Mitochondrial dysfunction resulting from loss of cytochrome c impairs cellular oxygen sensing and hypoxic HIF-alpha activation Cell Metab 2005 1 393 399 10.1016/j.cmet.2005.05.003 16054088 PMC3141219

[B60] Gasparre G Hervouet E de Laplanche E Demont J Pennisi LF Colombel M Mege-Lechevallier F Scoazec JY Bonora E Smeets R Smeitink J Lazar V Lespinasse J Giraud S Godinot C Romeo G Simonnet H Clonal expansion of mutated mitochondrial DNA is associated with tumor formation and complex I deficiency in the benign renal oncocytoma Hum Mol Genet 2008 17 986 995 18156159 10.1093/hmg/ddm371

[B61] Gasparre G Porcelli AM Bonora E Pennisi LF Toller M Iommarini L Ghelli A Moretti M Betts CM Martinelli GN Ceroni AR Curcio F Carelli V Rugolo M Tallini G Romeo G Disruptive mitochondrial DNA mutations in complex I subunits are markers of oncocytic phenotype in thyroid tumors Proc Natl Acad Sci USA 2007 104 9001 9006 10.1073/pnas.0703056104 17517629 PMC1885617

[B62] Gasparre G Romeo G Rugolo M Porcelli AM Learning from oncocytic tumors: Why choose inefficient mitochondria? Biochim Biophys Acta 1807 2010 633 642 10.1016/j.bbabio.2010.08.006 20732299

[B63] Kurelac I MacKay A Lambros MB Di Cesare E Cenacchi G Ceccarelli C Morra I Melcarne A Morandi L Calabrese FM Attimonelli M Tallini G Reis-Filho JS Gasparre G Somatic complex I disruptive mitochondrial DNA mutations are modifiers of tumorigenesis that correlate with low genomic instability in pituitary adenomas Hum Mol Genet 2013 22 226 238 10.1093/hmg/dds422 23049073

[B64] Drygin D Rice WG Grummt I The RNA polymerase I transcription machinery: an emerging target for the treatment of cancer Annu Rev Pharmacol Toxicol 2010 50 131 156 10.1146/annurev.pharmtox.010909.105844 20055700

[B65] Montanaro L Trere D Derenzini M Nucleolus, ribosomes, and cancer Am J Pathol 2008 173 301 310 10.2353/ajpath.2008.070752 18583314 PMC2475768

[B66] Chaika NV Yu F Purohit V Mehla K Lazenby AJ DiMaio D Anderson JM Yeh JJ Johnson KR Hollingsworth MA Singh PK Differential expression of metabolic genes in tumor and stromal components of primary and metastatic loci in pancreatic adenocarcinoma PLoS One 2012 7 e32996 10.1371/journal.pone.0032996 22412968 PMC3296773

[B67] Briston T Yang J Ashcroft M HIF-1alpha localization with mitochondria: a new role for an old favorite? Cell Cycle 2011 10 4170 4171 10.4161/cc.10.23.18565 22101263 PMC3272295

[B68] Guerra F Perrone AM Kurelac I Santini D Ceccarelli C Cricca M Zamagni C De Iaco P Gasparre G Mitochondrial DNA mutation in serous ovarian cancer: implications for mitochondria-coded genes in chemoresistance J Clin Oncol 2012 30 e373 378 10.1200/JCO.2012.43.5933 23150702

[B69] Gasparre G Porcelli AM Lenaz G Romeo G Relevance of mitochondrial genetics and metabolism in cancer development Cold Spring Harb Perspect Biol 2013 5 2 10.1101/cshperspect.a011411 PMC3552507 23378588

[B70] Schroedl C McClintock DS Budinger GR Chandel NS Hypoxic but not anoxic stabilization of HIF-1alpha requires mitochondrial reactive oxygen species Am J Physiol Lung Cell Mol Physiol 2002 283 L922 931 12376345 10.1152/ajplung.00014.2002

