# Supplementary figures and images for: Respiratory complex I is essential to induce a Warburg profile in mitochondria-defective tumor cells
Source: Cancer Metab. 2013 Mar 18;1:11. doi: 10.1186/2049-3002-1-11 (PMC4178211; doi:10.1186/2049-3002-1-11)

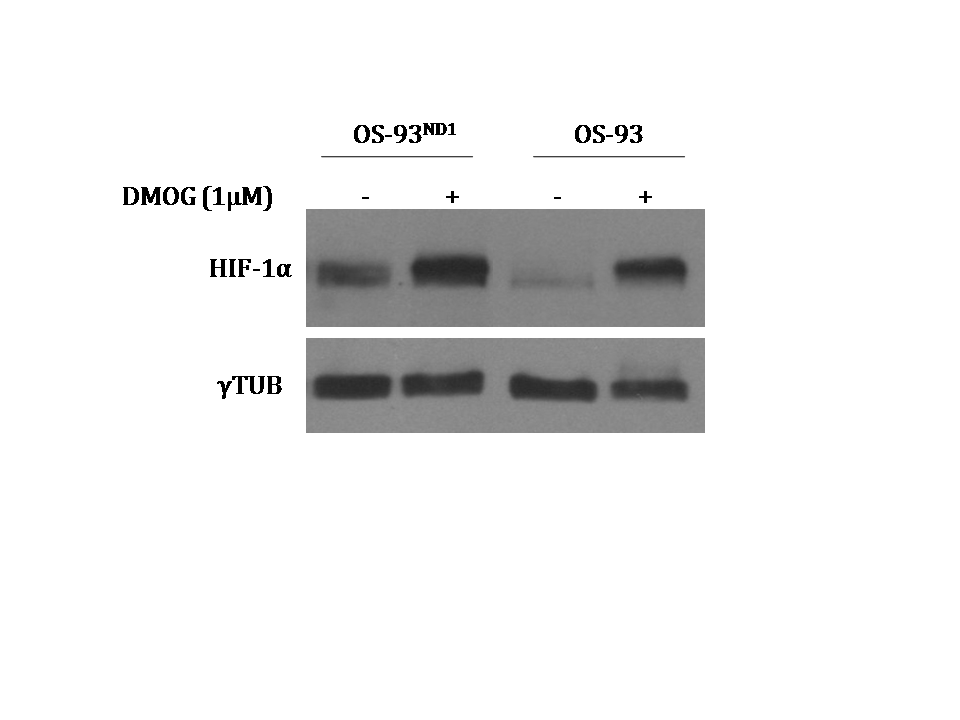

Supplement: Additional file 1: Figure S1 — DMOG effects validation. Western blot analysis for hypoxia inducible factor-1α (HIF-1α) protein on cell lysates upon treatment with 1 μM dimethyloxallylglycine (DMOG) indicate presence of stabilized HIF-1α only in the treated cells despite normoxic culture conditions. Tubulin was used as a loading control. [file 2049-3002-1-11-S1.tiff]

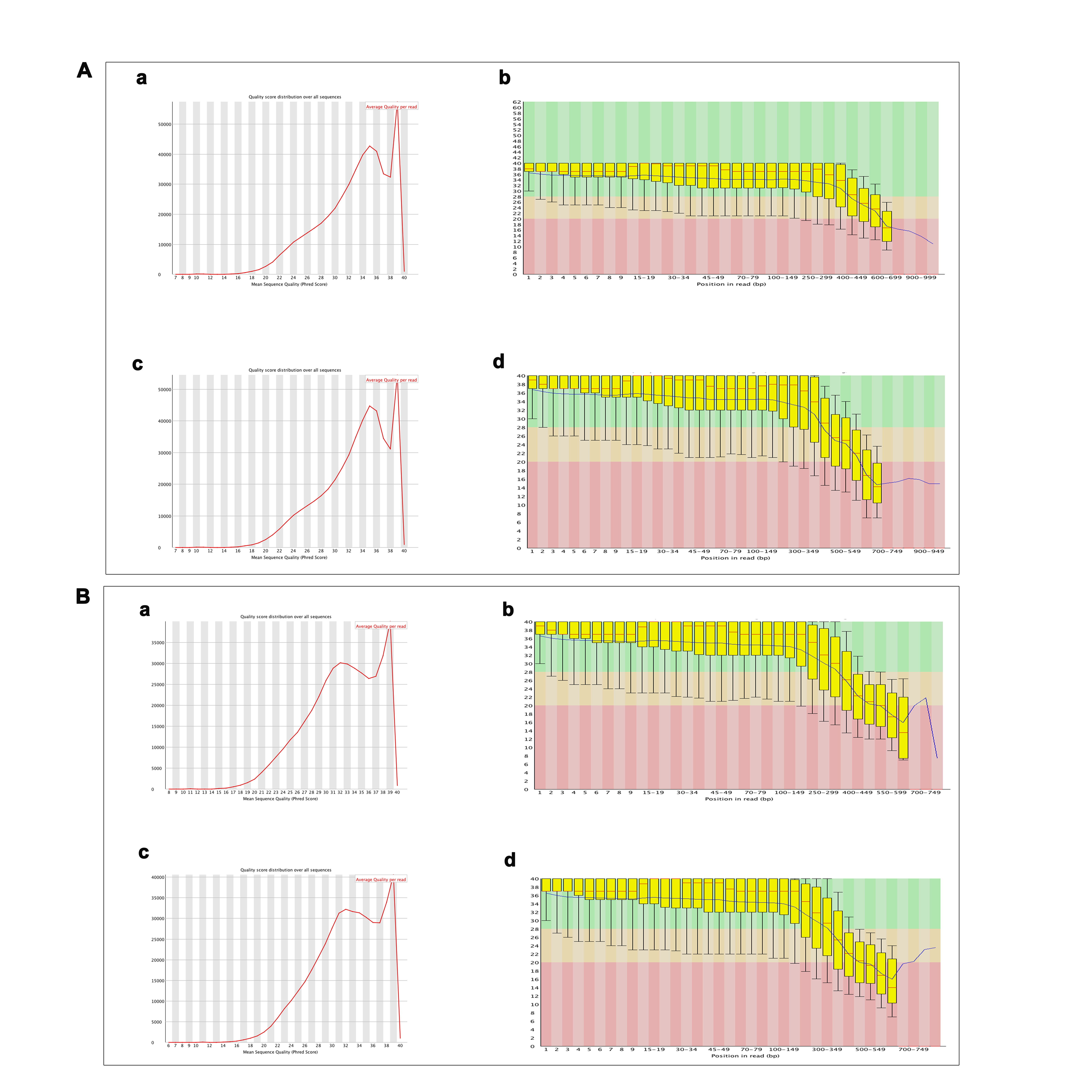

Supplement: Additional file 3: Figure S2 — FastQC analysis of fastq sequences from the four data sets of samples. Panel A and B refer respectively to OST-93 samples and to OST-93ND1 samples. (a-c) the distribution of the average quality scores per sequence within the set always ranges between 30 to 39 QS (quality score) values; (b-d) QS distribution per base position across the maximum read length observed within the data set. In all four samples the lower quartile associated to the 300th position of the read (average read length for each sequences data set) is always above 20. [file 2049-3002-1-11-S3.jpeg]

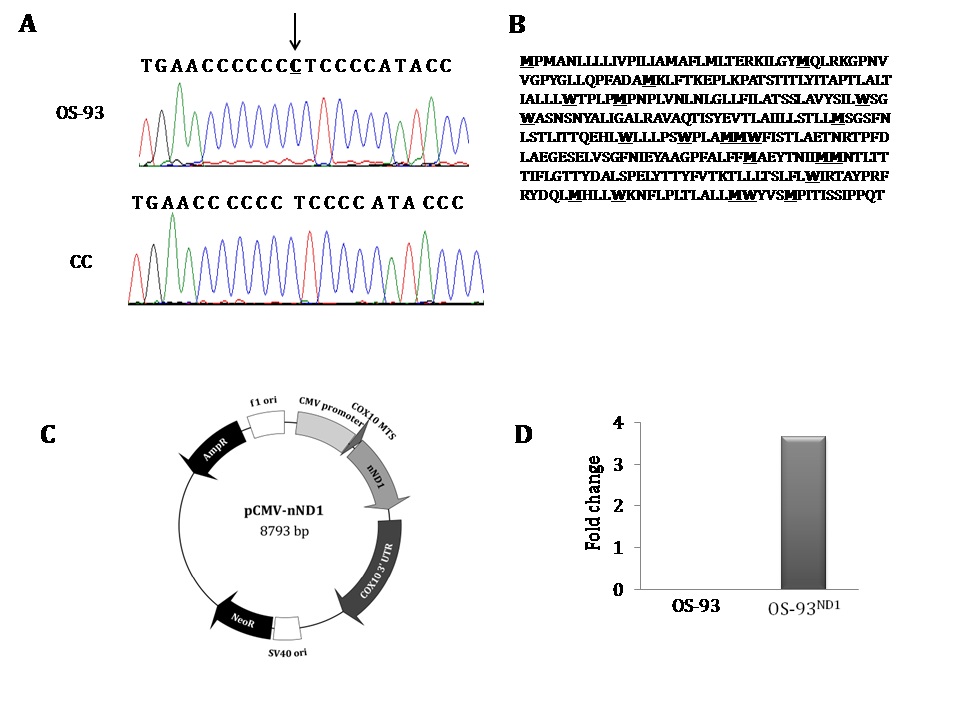

Supplement: Additional file 5: Figure S3 — Generation of allotopic nND1. (A) Elecropherogram showing m.3571insC in OS-93 cell line. (B) Amino acid sequence of ND1. The sites of directed mutagenesis performed to optimize codon usage for cytosolic translation of methionine (M) and tryptophan (W) are underlined and indicated in bold. (C) Scheme of the allotopic expression vector containing the nND1 transgene with the 3′- and 5′-UTR from the nuclear-encoded COX10 mitochondrial protein. Antibiotic resistance genes, CMV promoter, SV40 and f1 origin of replication are also indicated. (D) qRT-PCR showing mRNA expression of the cytosolically expressed nND1 construct in OS-93 cells. [file 2049-3002-1-11-S5.tiff]

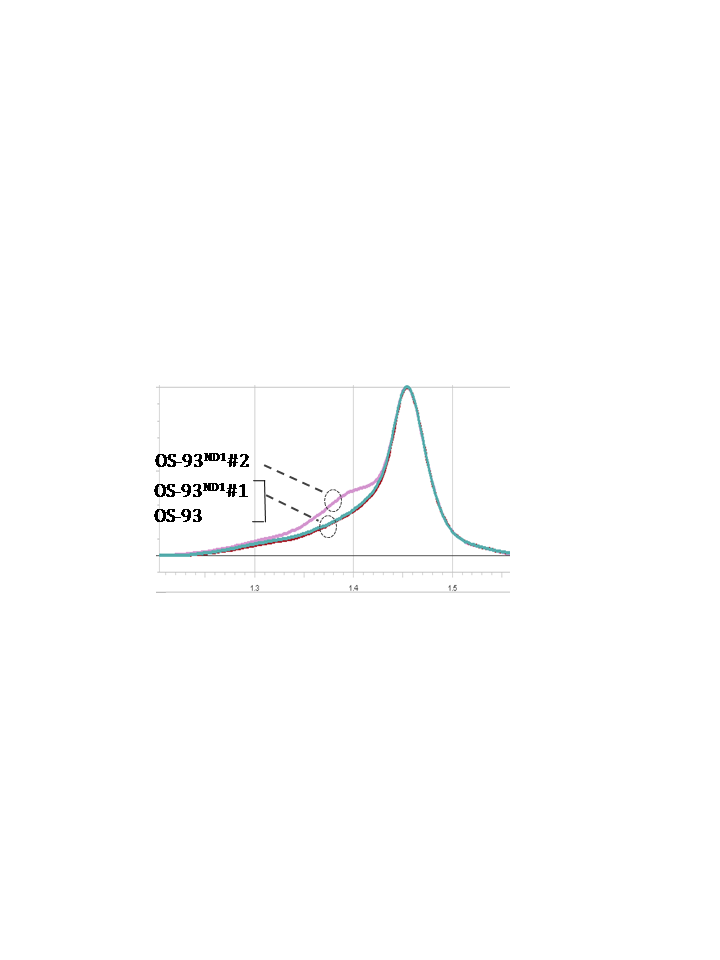

Supplement: Additional file 6: Figure S4 — Revertans exclusion. Denaturing high performance liquid chromatography (DHPLC) analysis of the allotopically complemented OS-93 cells. OS-93ND1#1 (cyan) has maintained the same m.3571insC mutant load as the original OS-93 cell line (red), while OS-93ND1#2 is a representative example of a revertant clone in which a higher proportion of wild-type molecules is evident from the elevated elution peak (pink). [file 2049-3002-1-11-S6.tiff]

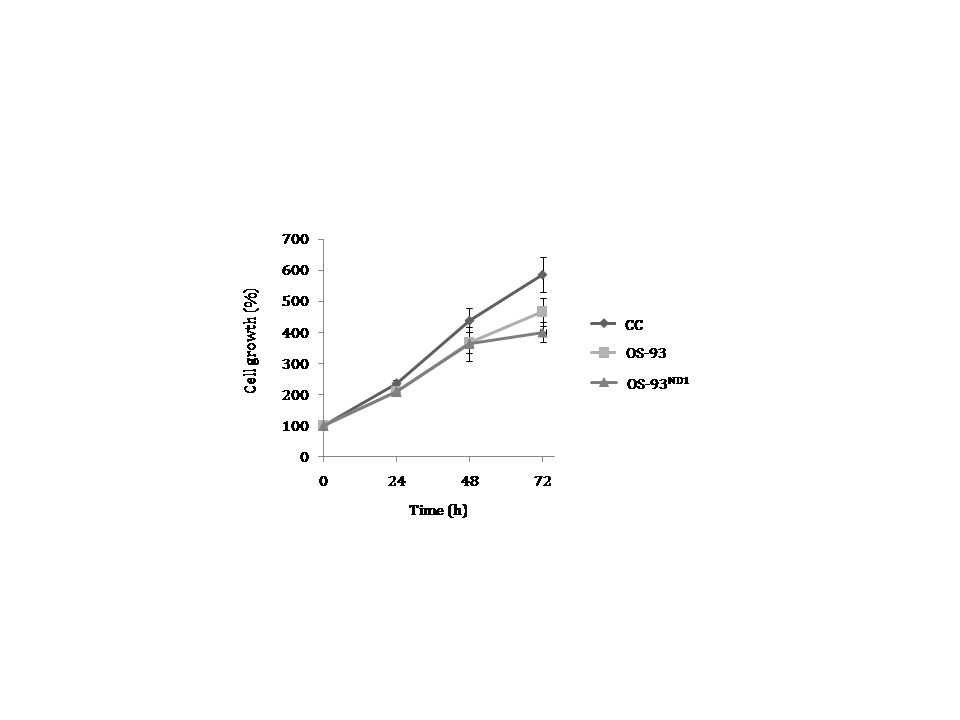

Supplement: Additional file 7: Figure S5 — Cellular growth determination. Cellular growth in DMEM-high glucose. Data represent mean ± standard error of the mean (n = 3). [file 2049-3002-1-11-S7.tiff]

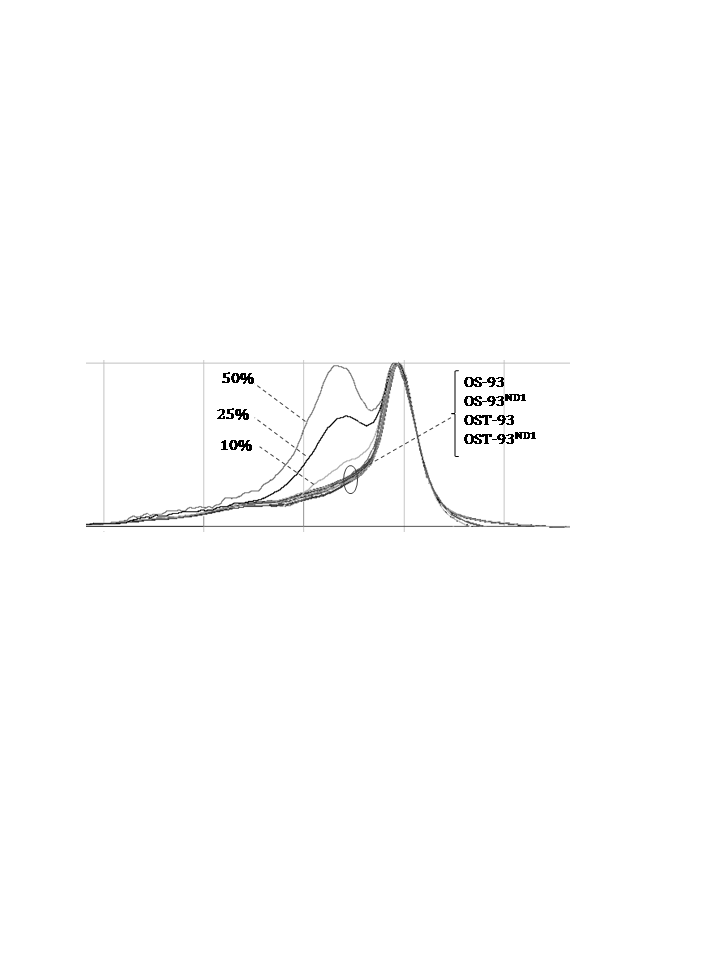

Supplement: Additional file 8: Figure S6 — Denaturing high performance liquid chromatography (DHPLC) analysis of OS-93 and OS-93ND1 clones and corresponding xenografts. Elution curves indicate maintenance of the m.3571insC genotype (mutant loads >90%). [file 2049-3002-1-11-S8.tiff]

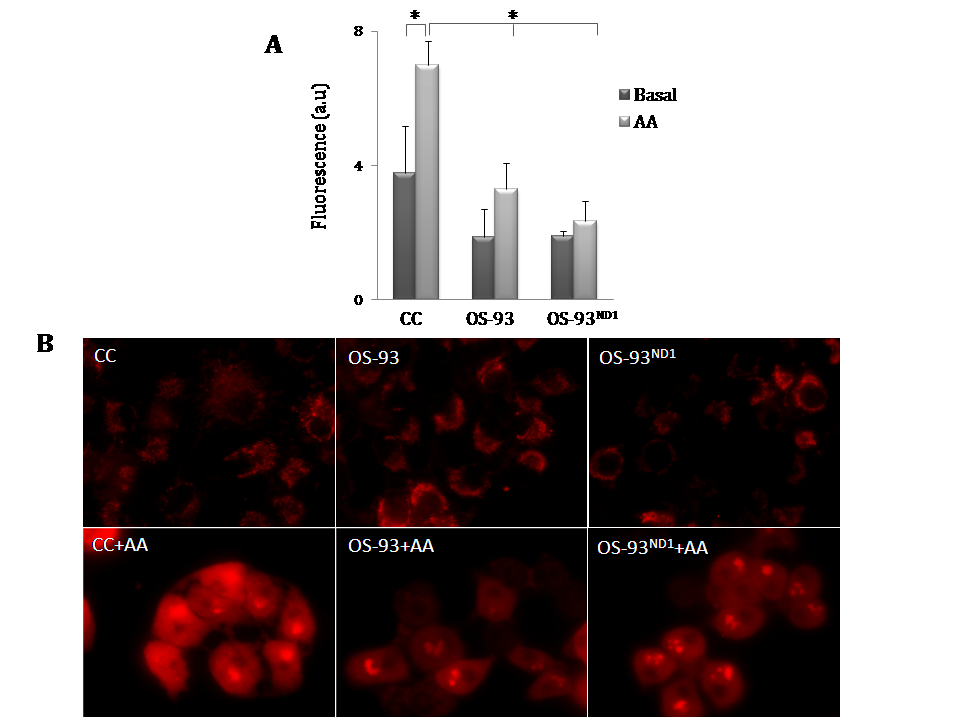

Supplement: Additional file 9: Figure S7 — Evaluation of reactive oxygen species (ROS) levels. (A) Hydrogen peroxide levels were measured using 2 μM H2DCFDA in the absence (basal) and presence of 1 uM antimycin A (AA) for 1 h, as described in Additional file 4; Methods. Data (mean ± SD, n = 3; *P <0.05) are expressed as ratio of fluorescence of H2DCFDA and calcein-AM. (B) Superoxide production was determined using 5 μM MitoSOX-Red, as described in Additional file 4; Methods. Images are representative of three different experiments. Magnification 63×/1.4. Ten images were acquired for each experiment. [file 2049-3002-1-11-S9.tiff]

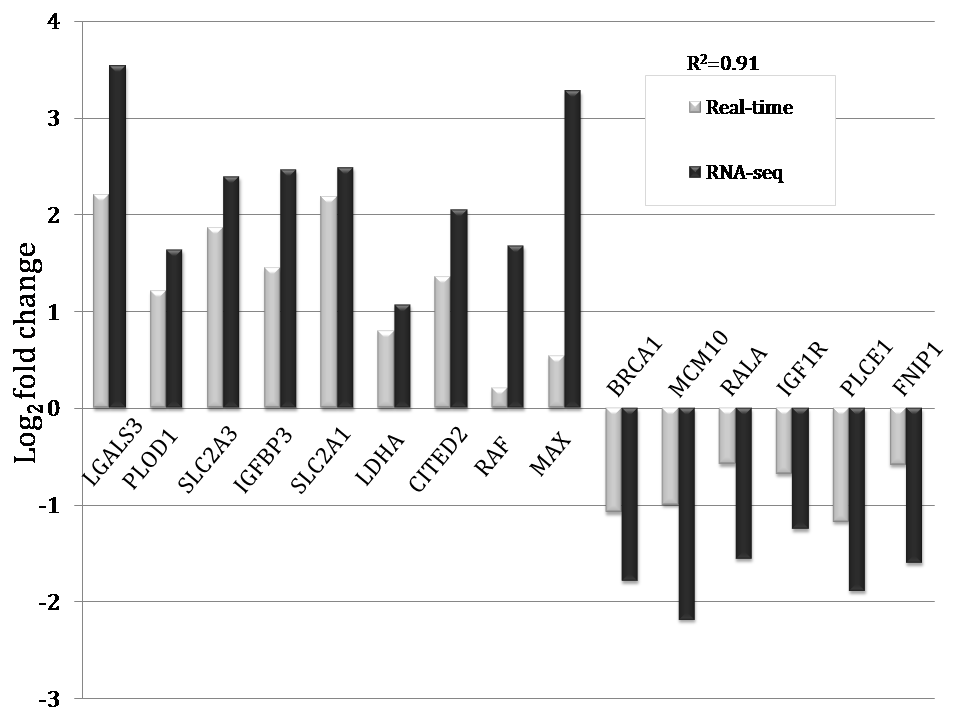

Supplement: Additional file 11: Figure S8 — Correlation between RNA-Seq analysis and qRT-PCR performed for 15/521 DE genes. Pearsons correlation coefficient was calculated using log2fold values. [file 2049-3002-1-11-S11.tiff]
