# Supplementary material for: Respiratory complex I is essential to induce a Warburg profile in mitochondria-defective tumor cells
Source: Cancer Metab. 2013 Mar 18;1:11. doi: 10.1186/2049-3002-1-11 (PMC4178211; doi:10.1186/2049-3002-1-11)
Supplement: Additional file 4 — Additional information. [file 2049-3002-1-11-S4.doc]

**Additional information**

1. **Additional Methods**
2. **Additional references**

**1. Additional Methods**

**Parameters for the NGS-TrEx mapping**

Reads were aligned on the human annotated genome hg18/NCBI_36 only if they were mapped for at least 70nt of their length and showed at least 95% of nucleotide identity with the genomic gene-coding region. To assign the read to a specific gene within the annotation step, 50nt of overlapping between each read and the genomic region annotated as ‘Genic’ were required, with at least 95% of nucleotide identity between the read and the annotated mRNA to assign it to a specific splicing isoform. A maximum trim of 15nt from the read’s edges was allowed in order to map the read onto the mRNA sequence. A less stringent *multiple mapping* approach was chosen in order to obtain high gene coverage results

**Differential expression analysis and functional categories enrichment**

Coverages per genic region with at least one read mapped were downloaded from NGS-TrEx platform and used as *digital expression value* for the differential expression analysis. In order to eliminate compositional biases of the two cDNA libraries compared in the experiment, the *CalcNormFactors* function of the *edgeR* package was used to calculate each normalization factors per sample, used to recalculate each library size value (i.e. total number of reads mapped onto genes per sample * normalization factor). Fold changes of gene expression were calculated by *edgeR* as ratio of digital expression values (OST-93^ND1^/OST-93). The *common dispersion* method was used to calculate the over-dispersion among the two samples compared in the experiment. The *Random sampling* method of the *goseq* package was used for functional Gene Ontology and KEGG categories enrichment analysis among all the 521 differentially expressed genes. Gene Ontology categories enrichments among each set of only over-expressed genes per group were conducted with the GeneMania software, assuming a network-weighting strategy based on query genes.

**Heatmaps construction**

The *maPalette* function from the *marray* R package was used to define heatmaps colors, while the *heatmap* R function was used to draw heatmaps. Values (digital gene expression) composing the input data matrix of the *heatmap* function were calculated as log2(n_ig_), where *n* is the total number of reads mapped onto the gene *g* for sample *i*.

**Validation of RNA-Seq data with qRT PCR**

Validation of RNA-Seq data was performed on cDNA (1:50 dilution) prepared by the retrotranscription of 1µg of total RNA with High Capacity cDNA Reverse Transcription Kit (Applied Biosystems) using random hexamers. Primer sequences were designed considering the exon regions covered by the reads in RNA-Seq and using Primer3 software (Rozen and Skaletsky, 2000). The presence of 3’ intra/inter primer homology was ruled out using IDT OligoAnalyzer tool (http://eu.idtdna.com/analyzer/Applications/OligoAnalyzer/) and SNP positions were verified based on the RNA-Seq data. The availability of the target sequence was evaluated by prediction of the cDNA secondary structure using Mfold web server (Zuker, 2003). Primer sequences and concentrations used in the qRT-PCR, together with the primer-specific PCR efficiencies are listed in Table S3. The PCR reaction was performed with GoTaq® qPCR Master Mix (Promega) and run in 7500 Fast Real-Time PCR System (Applied Biosystems), using following conditions: 95°C 5min; 45 cycles of 95°C 15sec and 60°C 45sec. The calculations were performed following 2^-ΔΔCT^ method. The normalization was performed with geometric average of the 2^ΔCT^ of two reference genes, namely *U2AF2* and *H2AFV*, which were selected based on RNA-Seq data. The statistical significance was calculated using the Δct values [ct(gene of interest)-ct(reference gene)] for each biological replicate in a group and applying T-test (Yuan et al., 2006).

**Cellular growth determination**

Cells (3x10^4^) were plated in DMEM high glucose and cellular growth was assessed at different time by using the colorimetric sulforhodamine B assay as previously described

**Determination of ROS production**

Hydrogen peroxide and anion superoxide production were measured in different cell clones after incubation respectively with 2μM of 2,7-dichlorodihydrofluorescein diacetate (H_2_DCFDA) and with 5μM of MitoSOX-Red (Molecular Probes, Invitrogen, Milan, Italy) in absence and in presence of 1µM antimycin A (AA) for 1h, as previously described. For hydrogen peroxide determination, cells were load with 1µM calcein-AM used as normalizer of adherent cells. H_2_DCFDA and calcein-AM fluorescence were determined at 535 and 488nm, respectively, using a multilabel counter Wallac 1420 (PerkinElmer, Turku, Finland). MitoSOX-Red fluorescence was visualized at 580nm by using a digital imaging system using an inverted epifluorescence microscope with 63X/1.4 oil objective (Nikon Eclipse Ti-U, Nikon, Japan). Images were captured with a back-illuminated Photometrics Cascade CCD camera system (Roper Scientific, Tucson, AZ, USA) with 500ms of exposure time and elaborated with Metamorph acquisition/analysis software (Universal Imaging Corp., Downingtown, PA, USA).

**2. Additional Tables**

**Additional Table 1. (separate Excel File)** Raw sequencing data together with the 521 genes differentially expressed among the two groups of samples (OST-93, OST-93^ND1^).

**Additional Table 2. (separate Excel File)** Gene Ontology and KEGG categories enrichment results. 34 GO categories and 8 KEGG categories were found significantly enriched among all the 521 differentially expressed genes. GO enrichments were also found by the GeneMania software among the two sets of only over-expressed genes per sample (226 up-regulated genes within the OST-93 group and 296 up-regulated genes within the OST-93^ND1^ group).

**Additional Table S3.** qRT-PCR primer sequences. (bp - base pairs; conc. - concentration; HIF-1α - hypoxia induced factor alpha; Eff. - PCR efficiency)

|  | Name | ENTREZ Gene ID | Sequence | Product length (bp) | Conc. in PCR (µM) | Slope | Eff. |
| --- | --- | --- | --- | --- | --- | --- | --- |
| HIF1α responsive genes | LGALS3_F | 3958 | GCCTTATAACCTGCCTTTGC | 142 | 0.2 | -3.398 | 97% |
|  | LGALS3_R |  | GAAGCGTGGGTTAAAGTGGA |  | 0.4 |  |  |
|  | PLOD1_F | 5351 | AGAAGCACGCAGACAAGGAG | 132 | 0.2 | -3.418 | 96% |
|  | PLOD1_R |  | CACCACCGGATACTTGGTCT |  | 0.4 |  |  |
|  | MCM10_F | 55388 | TGGGAACCTGTAAAGCCAAG | 150 | 0.2 | -3.463 | 94% |
|  | MCM10_R |  | CTCCAGAGAAGGTGGACTGC |  | 0.4 |  |  |
|  | SLC2A1_F | 6513 | ACTCCATCATGGGCAACAAG | 222 | 0.2 | -3.363 | 98% |
|  | SLC2A1_R |  | TCTGCCGACTCTCTTCCTTC |  | 0.4 |  |  |
|  | LDHA_F | 3939 | TGGGAGTTCACCCATTAAGC | 182 | 0.4 | -3.397 | 96% |
|  | LDHA_R |  | AGCACTCTCAACCACCTGCT |  | 0.4 |  |  |
|  | SLC2A3_F | 6515 | TCCACGCTCATGACTGTTTC | 111 | 0.2 | -3.269 | 102% |
|  | SLC2A3_R |  | GCCTGGTCCAATTTCAAAGA |  | 0.4 |  |  |
|  | BRCA1_F | 672 | GCATCTGGGTGTGAGAGTGA | 137 | 0.4 | -3.437 | 95% |
|  | BRCA1_R |  | AGTTCAGCCATTTCCTGCTG |  | 0.2 |  |  |
|  | CITED2_F | 10370 | GTCCCCTCTATGTGCTGCTG | 106 | 0.2 | -3.454 | 95% |
|  | CITED2_R |  | CATATGGTCTGCCATTTCCA |  | 0.4 |  |  |
|  | IGFBP3_F | 3486 | ACAGCCAGCGCTACAAAGTT | 154 | 0.6 | -3.341 | 99% |
|  | IGFBP3_R |  | CTGGGACTCAGCACATTGAG |  | 0.6 |  |  |
| Additional validation targets | MAX_F | 4149 | ATGCGAAGGAAAAACCACAC | 158 | 0.4 | -3.366 | 98% |
|  | MAX_R |  | GCGTTGGTGTAGAGGCTGTT |  | 0.2 |  |  |
|  | RALA_F | 5898 | CAGAGCTGAGCAGTGGAATG | 111 | 0.2 | -3.402 | 97% |
|  | RALA_R |  | TCCATCTTTCTCGCTCGAAT |  | 0.4 |  |  |
|  | RAF1_F | 5894 | TGGAGTCCCAGCACTACCTT | 135 | 0.4 | -3.332 | 99% |
|  | RAF1_R |  | CCTTCAGATGAGGGACTGGA |  | 0.2 |  |  |
|  | IGF1R_F | 3480 | ATTGCATGGTAGCCGAAGAT | 143 | 0.2 | -3.482 | 94% |
|  | IGF1R_R |  | TCCATCCTTGAGGGACTCAG |  | 0.4 |  |  |
|  | PLCE1_F | 51196 | TGAAGGATAAAAGCCGATGG | 116 | 0.4 | -3.72 | 98% |
|  | PLCE1_R |  | GGCCTTCTGTGAGTCCTCTG |  | 0.4 |  |  |
|  | FNIP1_F | 96459 | GGTGCTCTTCTGATGCCAAT | 123 | 0.6 | -3.49 | 93% |
|  | FNIP1_R |  | CTTTGCTGAGCATGAGCTGT |  | 0.4 |  |  |
| Reference genes | U2AF2_F | 11338 | CAGTGTTGGCTGTGCAGATT | 132 | 0.6 | -3.254 | 103% |
|  | U2AF2_R |  | GCCTGCGGATCTTTAGTGAC |  | 0.4 |  |  |
|  | H2AFV_F | 94239 | CGTGGTGATGAAGAGTTGGA | 154 | 0.2 | -3.472 | 94% |
|  | H2AFV_R |  | GCTCAGCACACATCCCAGTA |  | 0.4 |  |  |

1. **Additional Figure Legends**

**Additional Figure 1. DMOG effects validation.** Western blot analysis for HIF-1α protein on cell lysates upon treatment with 1μM DMOG indicate presence of stabilized HIF-1α only in the treated cells despite normoxic culture conditions. Tubulin was used as a loading control.

**Additional Figure 2. FastQC analysis of fastq sequences from the four data sets of samples.** Panel A and B refer respectively to OST-93 samples and to OST-93^ND1^ samples. **(a-c)** the distribution of the average quality scores per sequence within the set always ranges between 30 -39 QS (quality score) values; **(b-d)** quality score distribution per base position across the maximum read length observed within the data set. In all the four samples the lower quartile associated to the 300^th^ position of the read (average read length for each sequences data set) is always above 20.

**Additional Figure 3. Generation of allotopic *nND1*. (A)** Elecropherogram showing m.3571insC in OS-93 cell line. **(B)** Amino acid sequence of ND1. The sites of directed mutagenesis performed to optimize codon usage for cytosolic translation of Methionine (M) and Tryptophan (W) are underlined and indicated in bold. **(C)** Scheme of the allotopic expression vector containing the *nND1* transgene with the 3’- and 5’-UTR from the nuclear-encoded COX10 mitochondrial protein. Antibiotic resistance genes, CMV promoter, SV40 and f1 origin of replication are also indicated. **(D)** qRT-PCR showing mRNA expression of the cytosolically expressed *nND1* construct in OS-93 cells.

**Additional Figure 4. Revertans exclusion.** DHPLC analysis of the allotopically complemented OS-93 cells. OS-93^ND1#1^ (cyan) has maintained the same m.3571insC mutant load as the original OS-93 cell line (red), while OS-93^ND1#2^ is a representative example of a revertant clone in which higher proportion of wild-type molecules is evident from the elevated elution peak (pink).

**Additional Figure 5. Cellular growth determination.** Cellular growth in DMEM high glucose. Data represent mean±SEM (n =3).

**Additional Figure 6 DHPLC analysis of OS-93 and OS-93^ND1^ clones and corresponding xenografts.** Elution curves indicate maintenance of the m.3571insC genotype (mutant loads >90%).

**Additional Figure 7. ROS levels evaluation.** **(A)** Hydrogen peroxide levels were measured using 2μM H_2_DCFDA in absence (basal) and presence of 1uM AA (AA) for 1h, as described in additional methods. Data (mean±SD, *n*=3; *P<0.05) are expressed as ratio of fluorescence of H_2_DCFDA and calcein-AM. **(B)** Superoxide production was determined using 5μM MitoSOX-Red, as described in additional methods. Images are representative of 3 different experiments. Magnification: 63X/1.4. Ten images were acquired for each experiment.

**Additional Figure 8. Correlation between RNA-Seq analysis and qRT-PCR performed for 15/521 DE genes. Pearsons** correlation coefficient was calculated using log_2_fold values.

1. **Additional Information references**

Gasparre G, Kurelac I, Capristo M, Iommarini L, Ghelli A, Ceccarelli C, Nicoletti G, Nanni P, De Giovanni C, Scotlandi K *et al*: A mutation threshold distinguishes the antitumorigenic effects of the mitochondrial gene MTND1, an oncojanus function. *Cancer Res* 2011, 71(19):6220-6229.

Koopman WJ, Nijtmans LG, Dieteren CE, Roestenberg P, Valsecchi F, Smeitink JA, Willems PH: **Mammalian mitochondrial complex I: biogenesis, regulation, and reactive oxygen species generation**. *Antioxid Redox Signal* 2010, **12**(12):1431-1470.

Porcelli AM, Ghelli A, Iommarini L, Mariani E, Hoque M, Zanna C, Gasparre G, Rugolo M. (2008) [The antioxidant function of Bcl-2 preserves cytoskeletal stability of cells with defective respiratory complex I.](http://www.ncbi.nlm.nih.gov/pubmed/18695940) Cell Mol Life Sci. Sep;65(18):2943-51. doi: 10.1007/s00018-008-8300-2.

Rozen, S., and Skaletsky, H. (2000). Primer3 on the WWW for general users and for biologist programmers. Methods Mol Biol *132*, 365-386.

Zuker, M. (2003). Mfold web server for nucleic acid folding and hybridization prediction. Nucleic Acids Res *31*, 3406-3415.

Yuan, J. S., Reed, A., Chen, F., and Stewart, C. N., Jr. (2006). Statistical analysis of real-time PCR data. BMC Bioinformatics *7*, 85.
